# A hybrid variational autoencoder and WGAN with gradient penalty for tertiary protein structure generation

**DOI:** 10.1038/s41598-025-94747-y

**Published:** 2025-04-23

**Authors:** Aalaa I. Sehsah, Afaf Mousa, Gamal Farouk

**Affiliations:** 1 Department of Computer Science, Faculty of Computers and Information, Kafrelsheikh University, Kafr El Sheikh, 33516 Egypt; 2 Department of Computer Science, Faculty of Computers and Information, Menoufia University, Shebin El Kom, 32511 Egypt

**Keywords:** Tertiary protein structure, Residual blocks, VAE, WGAN-GP, Computational biology and bioinformatics, Machine learning

## Abstract

Elucidating the tertiary structure of proteins is important for understanding their functions and interactions. While deep neural networks have advanced the prediction of a protein’s native structure from its amino acid sequence, the focus on a single-structure view limits understanding of the dynamic nature of protein molecules. Acquiring a multi-structure view of protein molecules remains a broader challenge in computational structural biology. Alternative representations, such as distance matrices, offer a compact and effective way to explore and generate realistic tertiary protein structures. This paper presents TP-VWGAN, a hybrid model to improve the realism of generating distance matrix representations of tertiary protein structures. The model integrates the probabilistic representation learning of the Variational Autoencoder (VAE) with the realistic data generation strength of the Wasserstein Generative Adversarial Network with Gradient Penalty (WGAN-GP). The main modification of TP-VWGAN is incorporating residual blocks into its VAE architecture to improve its performance. The experimental results show that TP-VWGAN with and without residual blocks outperforms existing methods in generating realistic protein structures, but incorporating residual blocks enhances its ability to capture key structural features. Comparisons also demonstrate that the more accurately a model learns symmetry features in the generated distance matrices, the better it captures key structural features, as demonstrated through benchmarking against existing methods. This work moves us closer to more advanced deep generative models that can explore a broader range of protein structures and be applied to drug design and protein engineering. The code and data are available at https://github.com/aalaa-sehsah/tp-vwgan.

## Introduction

Research on protein tertiary structure prediction has helped us understand biological functions and potential impairments in living cells^1^, thus advancing fields such as drug design^2^, molecular inpainting^3^, protein classification^4^, and biocatalysis^5^. Determining the tertiary structure of proteins through methods such as X-ray crystallography^6^, cryogenic electron microscopy (cryo-EM)^7^, and nuclear magnetic resonance (NMR) spectroscopy^8^ is highly accurate but comes with significant challenges. X-ray crystallography requires protein crystallization, which is not feasible for all exceptionally flexible or membrane-bound proteins. Cryo-EM works well on large protein complexes, but it has a problem when it comes to small proteins. NMR, on the other hand, is a technique limited to smaller proteins because of size constraints. Therefore, not all protein structures can be determined by laboratory techniques, which show the need for computational methods to address these gaps.

Computational approaches are categorized into template-based and template-free modeling^9^. Template-based modeling predicts target structures by referencing related protein structures, while template-free modeling, often using de novo methods, predicts structures without prior templates. Recent advancements in computational models aim to address the challenges of both approaches, offering more efficient and accurate solutions for protein structure prediction. Among these advancements, state-of-the-art deep neural networks have shown their ability to predict a highly accurate tertiary structure of a protein from its amino acid sequence; Deep Mind’s AlphaFold2^10^ is a leading example, recognized as a solution to a 50-year grand challenge.

Recently, AlphaFold3^11^ has extended AlphaFold2’s exceptional accuracy in predicting protein structures by enabling the prediction of interactions between proteins and other molecules, such as DNA and RNA. This advancement broadens its applications in structural biology and enhances our understanding of molecular interactions.

Relying solely on the amino acid sequence to predict the protein structure does not account for the fact that proteins are intrinsically dynamic and can adapt their structure to interact with other molecules in the cell^12^ and because of this dynamic nature, obtaining a multi-structure view of protein molecules remains a continuing challenge.

The remaining challenges in capturing the multi-structured view of proteins and rapid advances in deep learning are currently driving interest in deep generative models as alternative frameworks^13^. Deep generative frameworks can learn multi-view representations of protein structures from structural biology repositories, such as the Protein Data Bank (PDB)^14^ and Structural Classification of Proteins (SCOP)^15^. Prominent examples of deep generative models used for generating protein structures and learning their complex representations include Generative Adversarial Networks (GANs)^16^ and Variational Autoencoders (VAEs)^17^.

VAEs rely on a probabilistic framework using an encoder-decoder structure. The encoder processes the input data into a compact latent space, represented as a probability distribution. The decoder then uses this latent representation to reconstruct the original data. This probabilistic approach helps VAEs to capture meaningful patterns, compress information efficiently, and generate new data samples. While VAEs are effective at capturing meaningful patterns and generating new data samples, they often face challenges in generating highly realistic samples, especially for complex data distributions.

Another type of generative framework is GANs, which employ a discriminator and a generator network that work adversarially to produce samples indistinguishable from real data. The discriminator evaluates whether a data point originates from the real data distribution or the generator, while the generator maps random noise into the data space to deceive the discriminator. However, standard GANs often encounter issues during training, such as instability and vanishing gradients. Wasserstein GAN with Gradient Penalty (WGAN-GP) addresses these challenges by replacing the discriminator with a critic, which measures the Wasserstein distance between real and generated data. By incorporating a gradient penalty, WGAN-GP ensures smooth and consistent updates during training, improving stability, enhancing gradient flow, and increasing model robustness^18^.

Hybrid models like VAEGAN integrate the strengths of VAEs with GANs, enabling the generation of realistic and diverse data, but VAEGAN struggles to balance reconstruction accuracy with adversarial training, which can affect its ability to create highly realistic samples^19^. These challenges make VAEGAN less suitable for handling complex data distributions, including protein distance matrices, which require precise structural details and stable training processes.

In this paper, we present TP-VWGAN, a hybrid deep generative model that improves the realism of generating distance matrix representations of tertiary protein structures. The model combines the probabilistic representation learning of the VAE with the adversarial training stability and realistic data generation strength of the WGAN-GP and includes residual blocks to enhance the model’s ability to capture key structural features of tertiary protein structures.

Protein distance matrices require models capable of maintaining accurate geometric relationships and capturing complex structural patterns, which traditional generative models often struggle to achieve. TP-VWGAN bridges this gap by combining probabilistic learning with stable adversarial training, enhanced by residual blocks, which allows it to generate distance matrices that are both realistic and diverse. This integration allows TP-VWGAN to address limitations often encountered in generative models, including VAE, GAN, WGAN-GP, and VAEGAN, in handling distance matrices, such as challenges in balancing reconstruction accuracy, adversarial objectives, and training stability. We also compare TP-VWGAN against prominent generative models used for the same task, using all respective evaluation metrics found in related work for fair comparison.

TP-VWGAN represents a significant advancement in deep generative frameworks by addressing specific challenges in generating accurate and diverse distance matrix representations for protein engineering. It also holds potential for drug design and structure prediction applications, providing valuable data augmentation to support interaction modeling and address gaps in structural data.

The paper’s main contributions are as follows:

1. This paper presents TP-VWGAN, which combines VAE and WGAN-GP to improve the realism of generating distance matrix representations for tertiary protein structures. The primary enhancement in its architecture is the incorporation of residual blocks into the VAE to improve its performance.
2. The validation proves that residual blocks improve the ability of TP-VWGAN to capture key structural features, as demonstrated by an ablation study comparing its performance with and without the residual blocks.
3. The comparisons prove that the more accurately a model learns symmetrical features in the generated distance matrices, the better it captures key structural features, as demonstrated through benchmarking across all evaluated models.
4. The experimental results prove that TP-VWGAN effectively captures key structural features and achieves higher symmetry than existing methods.

The structure of this paper is as follows: Sect. "Related work" introduces the related work and research gap. Section "Results" describes the results of our proposed approach. Section "Discussion of results" presents a discussion of our results. Section "Conclusion and future work" presents the conclusion and future work. Section "Methodology" presents methodological details and evaluation metrics.

## Related work

Recent works have used deep generative models to generate different representations of tertiary protein structures. The distance matrix is a common representation that summarizes the tertiary structure of a protein molecule of $\:N$ amino acids (aa) to an $\:N\times\:N$ distance matrix recording Euclidean pairwise distances between alpha carbon (Cα) atoms. This representation is very useful as it captures both local and distal spatial relationships that are important for understanding protein structure and dynamics. Compared to other representations, such as the contact map, a binary matrix derived from the distance matrix that shows contacts between residues if their Cα atoms are about 8 Å apart^20^, or graph-based representations where nodes represent residues and edges define structural relationships^21,22^, distance matrices provide a balance between computational simplicity and structural detail. This balance makes distance matrices important to understanding dynamic protein conformations, which show how proteins interact with other molecules, making them very useful for drug design, such as in identifying binding sites or generating alternative conformations for molecular docking analyses^23^. It also enables generative models to capture complex structural patterns and address challenges in protein modeling, such as predicting realistic tertiary structures, generating alternative conformations, and completing unresolved regions in experimental protein structures. The different representations of protein structures focus on specific features and allow deep generative models to address a variety of challenges in protein modeling.

Namarate et al.^24^ proposed a convolutional GAN to learn from Cα distance matrices using protein fragments with fixed lengths of 16, 64, or 128 aa obtained from experimentally available structures in the PDB. GAN aimed to explore structural diversity and realism in protein modeling but faced challenges capturing structural features, which highlights the need for better generative models to improve the realism of generated distance matrices.

Rahman et al.^25^ applied various mechanisms to improve the performance of GANs for modeling protein tertiary structures through distance matrix representations and introduced rigorous metrics to evaluate how well the model captures key tertiary structural features in distance matrices, including local and distal patterns (short-range and long-range contacts). While these mechanisms improved some aspects of GAN performance, they failed to effectively capture complex distal patterns and suffered from instability during training. To address these limitations, Rahman et al.^25^ utilized WGAN, which achieved a better balance in capturing both local and distal structural patterns, although challenges such as stability and generating highly realistic distance matrices remained.

Mena et al.^26^ introduced ROD-WGAN, which incorporated the Ratio of Distribution (ROD) to enhance the gradient penalty in WGAN-GP. Compared to WGAN by Rahman et al.^25^, ROD-WGAN showed better realism in generating distance matrices for tertiary protein structures but relied on metrics that average backbone, short-range, and long-range distances rather than the rigorous contact-based metrics proposed by Rahman et al.^25^ (further details in Sect. "Methodology"). ROD-WGAN faced challenges in accurately representing the real distribution of structural features and did not use rigorous evaluation metrics. Unlike WGAN and ROD-WGAN, which relied on separate evaluation approaches, TP-VWGAN integrates both contact-based and distance-based metrics, demonstrating greater accuracy in capturing real structural distributions.

GAN models have also shown their ability in loop modeling, which involves predicting missing regions in protein structures often unresolved in experimental methods. The first use of GANs for loop modeling in protein structures was introduced in^27,28^. Loop modeling can use different structural representations, but distance matrices are especially helpful for capturing spatial relationships, which makes it easier to build a complete and accurate protein model. Recently, Mena et al.^29^ proposed PLM-GAN, which is based on the Pix2Pix GAN framework^30^, to complete larger and more accurate regions in distance matrices.

Besides GAN-based models, VAEs offer a complementary approach to protein structure modeling, showing early promise in training on computationally generated protein structures. Degiacomi et al.^31^ utilized autoencoders trained on molecular dynamics simulations to generate plausible protein conformations, focusing on applications such as protein-protein docking. Alam et al.^32^ employed VAEs trained on protein structures obtained through Rosetta, concentrating on Cα atomic coordinates. While Degiacomi et al.^31^ and Alam et al.^32^ were successful in capturing the diversity of protein structures, both studies relied on computationally generated data for training and did not compare their results rigorously with experimental data.

Fardina et al.^33^ introduced a convolutional VAE with Spatial Pyramid Pooling (CVAE-SPP) designed to learn from experimentally available tertiary protein structures and demonstrated its ability to generate realistic tertiary structures from distance matrices. Spatial Pyramid Pooling enables the CVAE-SPP model to process distance matrices of varying sizes by generating fixed-size results while capturing key structural features, but its effectiveness depends on resolution settings and may introduce errors during post-processing. In a subsequent study, Fardina et al.^34^ explored the effects of data quality, size, and composition on the performance of generative models, showing that diverse and high-resolution training data improve the learning of both local and distal structural patterns. While the studies by Fardina et al.^33,34^ emphasized the importance of dataset design in generating realistic outputs, neither provided a direct comparison between the CVAE-SPP model and GAN-based architectures.

Hybrid models like VAEGAN enable the generation of realistic and diverse data, but VAEGAN struggles with balancing reconstruction accuracy and adversarial objectives, particularly for complex data distributions such as protein distance matrices. TP-VWGAN addresses these limitations by leveraging the stability of WGAN-GP and incorporating residual blocks, ensuring accurate and diverse distance matrix representations. This makes TP-VWGAN particularly well-suited for capturing geometric relationships and complex patterns in distance matrix representation.

### The research gap

Despite advances in deep generative modeling for distance matrix representations of protein tertiary structures, key challenges remain. GANs often face training instability and struggle to accurately capture the real distribution of structural features. A thorough comparison of VAEs and GAN-based models is needed to assess their strengths and weaknesses in generating distance matrices. Existing methods use different evaluation metrics, with some focusing on contact-based metrics and others on average distances between residues, which challenges consistent assessments of structural realism. The evaluation of the symmetry of distance matrices, which is a key property essential for accurate representations, is not considered.

## Results

This section provides a detailed evaluation of the TP-VWGAN model in two configurations: with residual blocks (TP-VWGAN w/ res) and without residual blocks (TP-VWGAN w/o res). The evaluation analyzes the model’s convergence during training, assesses the risk of overfitting, its ability to capture key structural features, and the alignment between generated and real distributions through quantitative and visual analyses, compared to WGAN_Rahman_^22^ and ROD-WGAN^23^. It also includes an ablation study to assess the impact of residual blocks on the model’s performance in learning structural features and aligning distributions.

### Convergence analysis

**Fig. 1:**
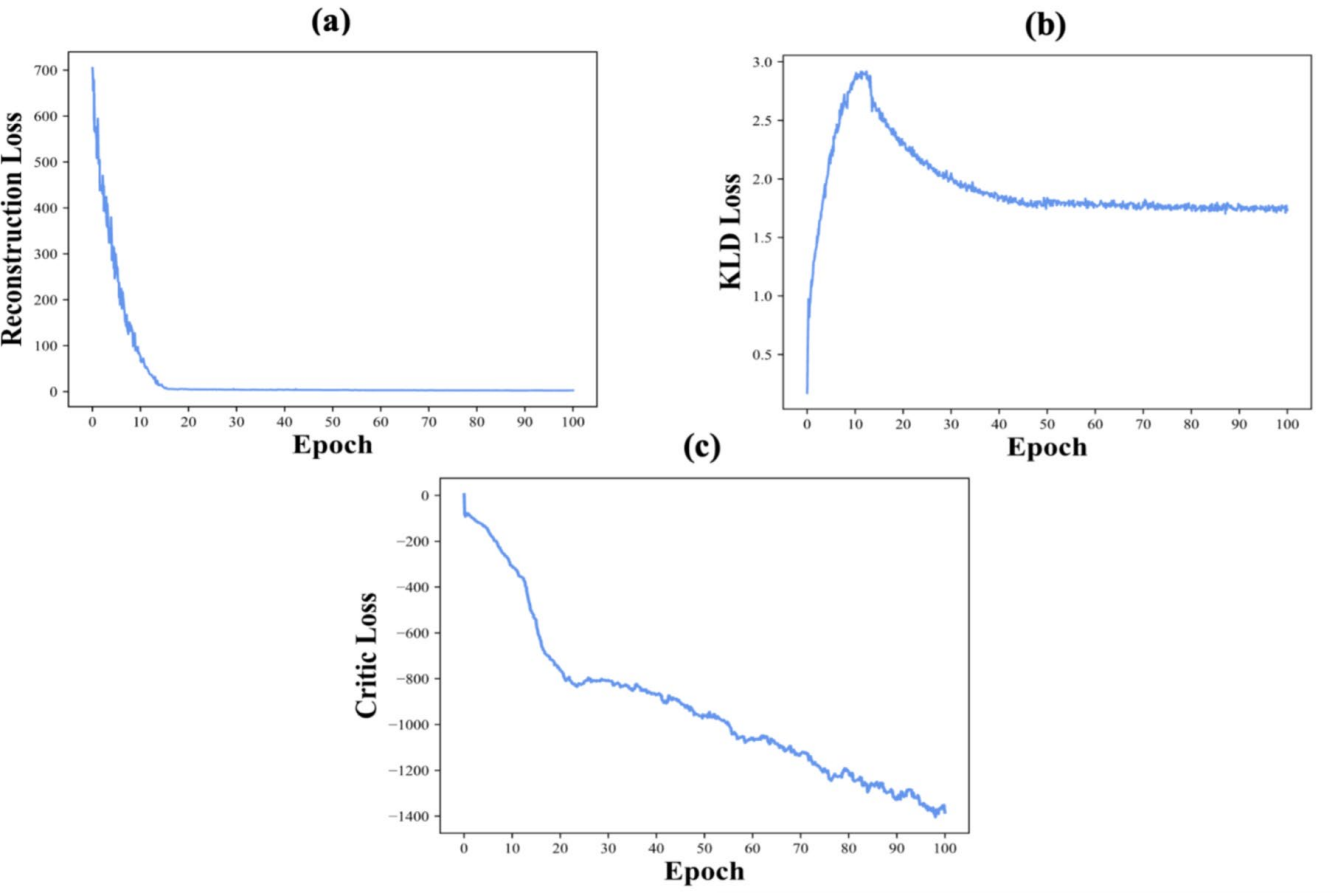
Training loss curves over 100 epochs: (**a**) Reconstruction loss, (**b**) KLD loss, and (**c**) Critic loss.

Figure 1 shows the training loss curves of the methodology over 100 epochs: reconstruction loss (panel a), KLD loss (panel b), and critic loss (panel c). The reconstruction loss declines rapidly during the first 20 epochs and then stabilizes, indicating that the decoder (generator) effectively learns to reconstruct input data and can generalize for generating realistic samples. KLD loss increases in the first epochs as the model optimizes the latent space, then stabilizes around epoch 20, which demonstrates a balance between reconstruction accuracy and latent space regularization.

The critical loss decreases throughout training and provides meaningful gradients to the generator, which avoids vanishing gradient issues. This consistent decrease means effective adversarial feedback and reliable training stability. The plots show that the methodology converges steadily, keeps training stable, and aligns the generated data closely with real distributions.

### Evaluation of overfitting

This subsection assesses the overfitting and generalization of TP-VWGAN using Contact Score, Root Mean Square Deviation (RMSD), and Template Modeling Score (TM-Score). Contact Score^35^ measures the similarity between two protein structures by comparing their residue-residue interaction patterns. These interactions are represented as contact maps, which are derived from distance matrices by using an 8-Å threshold to define residue pairs that are in contact. Contact Score is computed using the Jaccard Similarity Index, which quantifies the fraction of shared contacts between two maps. It ranges from 0 to 1, where higher values indicate greater similarity between structures. RMSD^36^ measures how much the distances between corresponding residues in two protein structures differ. Lower values indicate greater structural similarity. RMSD usually falls between 0 and over 10 Å, where values below 2 Å suggest nearly identical structures, values between 2 and 5 Å indicate moderate similarity, and values above 8 Å generally reflect significant structural differences. TM-Score^37^ evaluates the overall similarity between protein structures on a scale from 0 to 1. It is traditionally computed using atomic coordinates, but it assesses how well the global pairwise residue distance patterns are preserved between generated and real structures when adapted for distance matrices. Scores above 0.5 generally indicate similar global folding patterns, while scores below 0.3 suggest unrelated structures.

A smaller sample size was chosen to reduce computational cost while still capturing variability and diversity. A subset of 2,000 distance matrices generated by TP-VWGAN was compared with the entire training and testing datasets, and a subset of 2,000 randomly selected test samples was compared with the full training dataset.

The comparison between training and testing datasets had a mean Contact Score of 0.335 with a standard deviation of 0.108, confirming that they share fewer residue-residue contacts and have apparent structural differences. The comparatively higher standard deviation highlights the diversity in the test set, reinforcing that the testing dataset is not just a variant of the training data. The samples generated had a mean Contact Score of 0.426 when compared to the training data and 0.471 when compared to the testing data, with standard deviations of 0.060 and 0.075, respectively. Since the Contact Score for generated samples is higher against test data than training data, this confirms that the model does not replicate training structures but instead captures meaningful structural patterns present in unseen proteins. The difference in variability further supports this conclusion. The lower standard deviation in comparison with training data suggests that generated structures retain essential training features without excessive memorization, while the slightly higher standard deviation in comparison with test data indicates that the generated structures introduce a reasonable degree of variation, which ensures that they are not direct copies of the test set but still align with its structural diversity. The result of the Contact Score comparison demonstrates that TP-VWGAN generalizes beyond its training data while maintaining biologically meaningful structural relationships.

The RMSD analysis confirms that TP-VWGAN effectively generalizes while maintaining clear structural distinctions between training and testing datasets. The RMSD between training and testing datasets had a mean of 7.648 Å with a standard deviation of 1.859 Å, demonstrating significant structural differences and highlighting the diversity of protein structures in the test set. The generated samples had a mean RMSD of 5.823 Å when compared to the training data and 5.147 Å when compared to the testing data, with standard deviations of 1.124 Å and 1.057 Å, respectively. The fact that the RMSD for generated samples is lower against test data than training data confirms that the model does not simply replicate training structures but generates proteins that align more closely with unseen test structures while maintaining structural variability. The moderate standard deviation values further support this by ensuring that the generated samples introduce meaningful diversity without excessive deviation. These results of RMSD confirm that TP-VWGAN learns biologically relevant structural patterns and successfully generalizes to novel protein conformations beyond its training distribution.

**Fig. 2:**
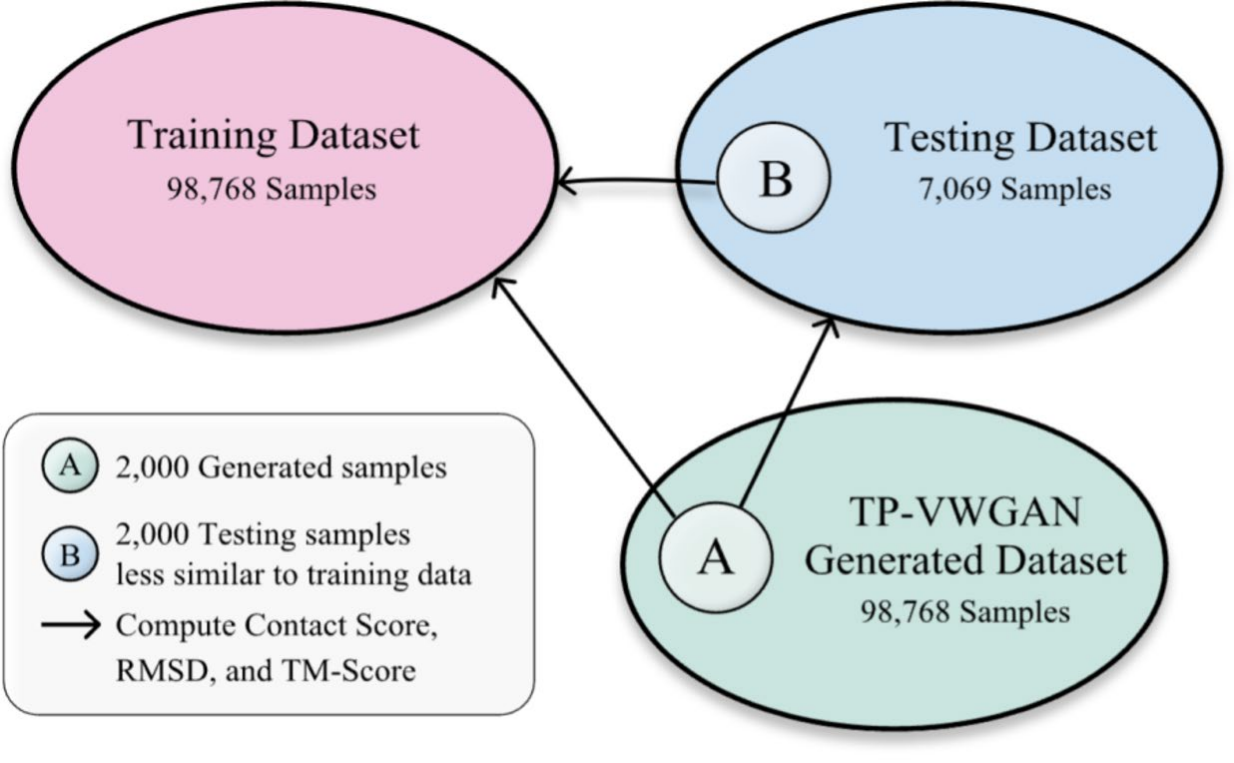
Evaluation overview of comparisons.

The TM-Score analysis further validates that TP-VWGAN effectively generalizes without overfitting. The TM-Score between training and testing datasets had a mean of 0.415 with a standard deviation of 0.081, reinforcing that the test set contains structurally distinct proteins compared to the training set. The generated samples had a mean TM-Score of 0.482 when compared to the training data and 0.528 when compared to the testing data, with standard deviations of 0.069 and 0.058, respectively. The fact that the generated samples align more closely with test structures than training structures confirms that the model does not simply memorize training data but successfully captures meaningful structural features of unseen proteins. The lower standard deviation for Generated vs. Test suggests that the model produces structurally consistent and biologically relevant outputs, while the moderate variation in Generated vs. Train further supports the claim that the model does not replicate training data. Together, these results confirm that TP-VWGAN successfully learns essential structural patterns without memorization, enabling it to generate diverse and biologically relevant protein conformations. Table 1 shows the evaluation of Overfitting Using Contact Score, RMSD, and TM-Score. Figure 2 also shows the evaluation overview of Comparisons. The images in Fig. 2 were created using Figma (Version 125.0.8)^38^.

**Table 1:** Evaluation of overfitting using contact score, RMSD, and TM-score.

	Statistics	Generated vs. train	Generated vs. test	Train vs. test
Contact score	Mean	0.426	0.471	0.335
	Std	0.060	0.075	0.108
RMSD (Å)	Mean	5.823	5.147	7.648
	Std	1.124	1.057	1.859
TM-score	Mean	0.482	0.528	0.415
	Std	0.069	0.058	0.081

### Structural feature evaluation

This subsection evaluated the structural fidelity of the generated distance matrices generated by TP-VWGAN w/ res, TP-VWGAN w/o res, ROD-WGAN, and WGAN_Rahman_. A total of 98,728 distance matrices were generated from each compared model at every 10th epoch. The evaluation used the metrics outlined in Sect. "Methodology" to thoroughly assess the presence of backbone, short-range, and long-range structural features.

#### Backbone structure evaluation

**Fig. 3:**
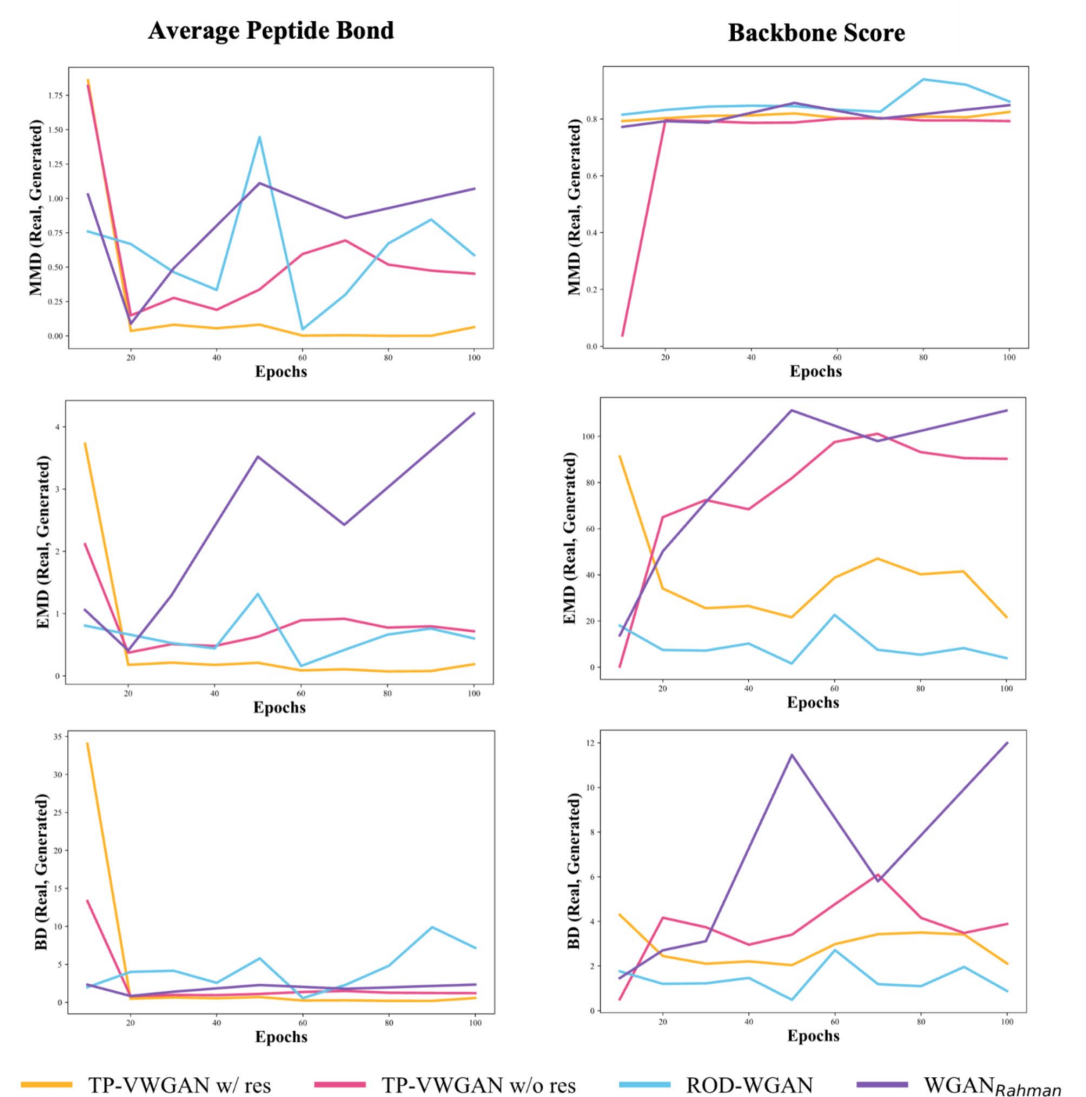
The comparison of the distributions of the backbone structure between real and generated distance matrices across models over epochs using MMD, EMD, and BD metrics.

Figure 3 compares the backbone structure of the generated distance matrices to real data distributions. The left column shows the Average Peptide Bond Length, and the right column shows the Backbone Score. The metrics MMD, EMD, and BD are organized into rows with lower values, meaning the generated data is closer to the real data.

The Average Peptide Bond (left column) shows that TP-VWGAN w/ res is closest to real data distribution with consistently lower MMD, EMD, and BD values. TP-VWGAN w/ res also performs well but has slightly higher metric values than TP-VWGAN w/ res. ROD-WGAN shows larger deviations, with weaker alignment to the real data, mainly in MMD and BD. WGAN_Rahman_ performs the worst, with much higher metric values that reflect reduced physical realism.

The Backbone Score (right column) shows an increase in MMD values across all models as training progresses, indicating a gradual divergence from the real data distribution. This increase in MMD may be attributed to the models generating more diverse backbone patterns, which slightly deviate from the real data. The models optimize multiple features, with less weight for the Backbone Score than the Average Peptide Bond Length, balancing diversity and structural alignment but causing slight deviations from real data. The EMD and BD metrics remain steady and show that the generated data maintains reasonable alignment for most models. Among the models, ROD-WGAN shows the closest alignment with the real data for the Backbone Score, followed by TP-VWGAN w/ res, which performs reliably but demonstrates a slight increase in MMD over time. TP-VWGAN w/o res and WGAN_Rahman_ show greater deviations across all metrics, indicating weaker performance in capturing the backbone structure than the other models.

#### Short-range structure evaluation

**Fig. 4:**
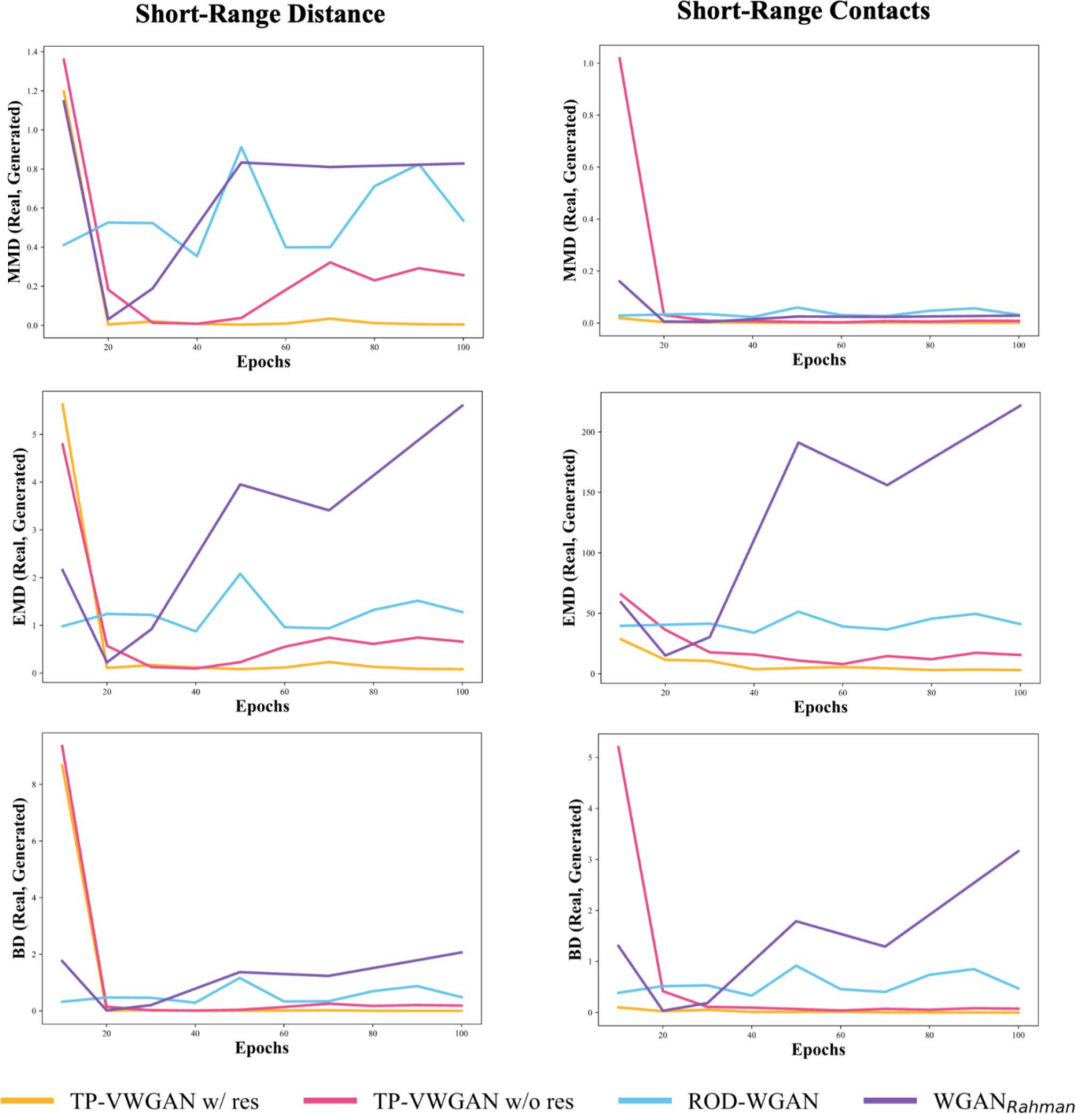
The comparison of the distributions of the short-range structure between real and generated distance matrices across models over epochs using MMD, EMD, and BD metrics.

Figure 4 evaluates the short-range structure in the generated distance matrices compared to real data distributions. The left column shows Short-Range Distance, and the right column shows Short-Range Contacts. Metrics MMD, EMD, and BD are displayed in separate columns.

The Short-Range Distance (left column) shows TP-VWGAN w/ res matches the real distribution best, with consistently low values for all metrics. TP-VWGAN w/o res performs well but has slightly higher deviations, especially in MMD. ROD-WGAN shows larger deviations and some fluctuations. WGAN_Rahman_ performs the worst, with the highest values for all metrics.

The Short-Range Contacts (right column) confirm that TP-VWGAN w/ res best aligns with the real data across all metrics. TP-VWGAN w/o res performs well but has slightly higher values than TP-VWGAN w/ res. ROD-WGAN has larger deviations and fluctuates more, particularly in EMD. WGAN_Rahman_ continues to perform poorly, with the highest and least stable values for all metrics.

#### Long-range structure evaluation

**Fig. 5:**
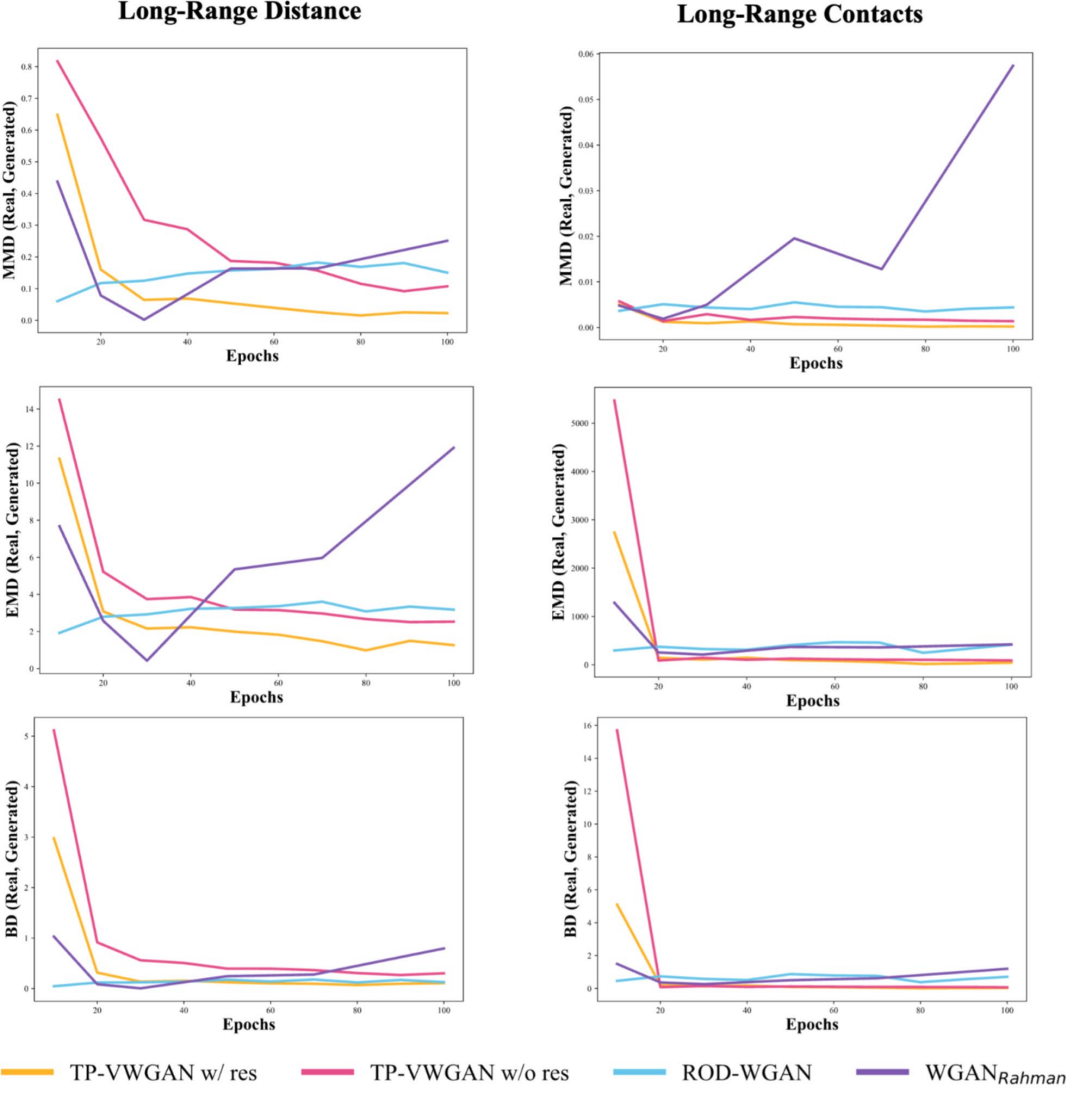
The comparison of the distributions of the long-range structure between real and generated distance matrices across models over epochs using MMD, EMD, and BD metrics.

Figure 5 evaluates the long-range structural features in the generated distance matrices compared to real data distributions. The left column represents Long-Range Distance, while the right column illustrates Long-Range Contacts. Metrics MMD, EMD, and BD are displayed in separate rows.

The Long-Range Distance (left column) shows that TP-VWGAN w/ res performs the best, with the lowest metric values. TP-VWGAN w/o res also performs well but has slightly higher deviations, especially in MMD and EMD. ROD-WGAN shows moderate results, with stable values but higher deviations than the TP-VWGAN models. WGAN_Rahman_ performs the worst with the highest values.

The Long-Range Contacts (right column) confirm TP-VWGAN w/ res again as the best model, with the lowest and most stable values. TP-VWGAN w/o res also performs well but has slightly higher deviations. ROD-WGAN shows moderate alignment, with stable metrics but higher values than TP-VWGAN models. WGAN_Rahman_ again performs the worst, with the highest and least stable values.

### Symmetry analysis

**Fig. 6:**
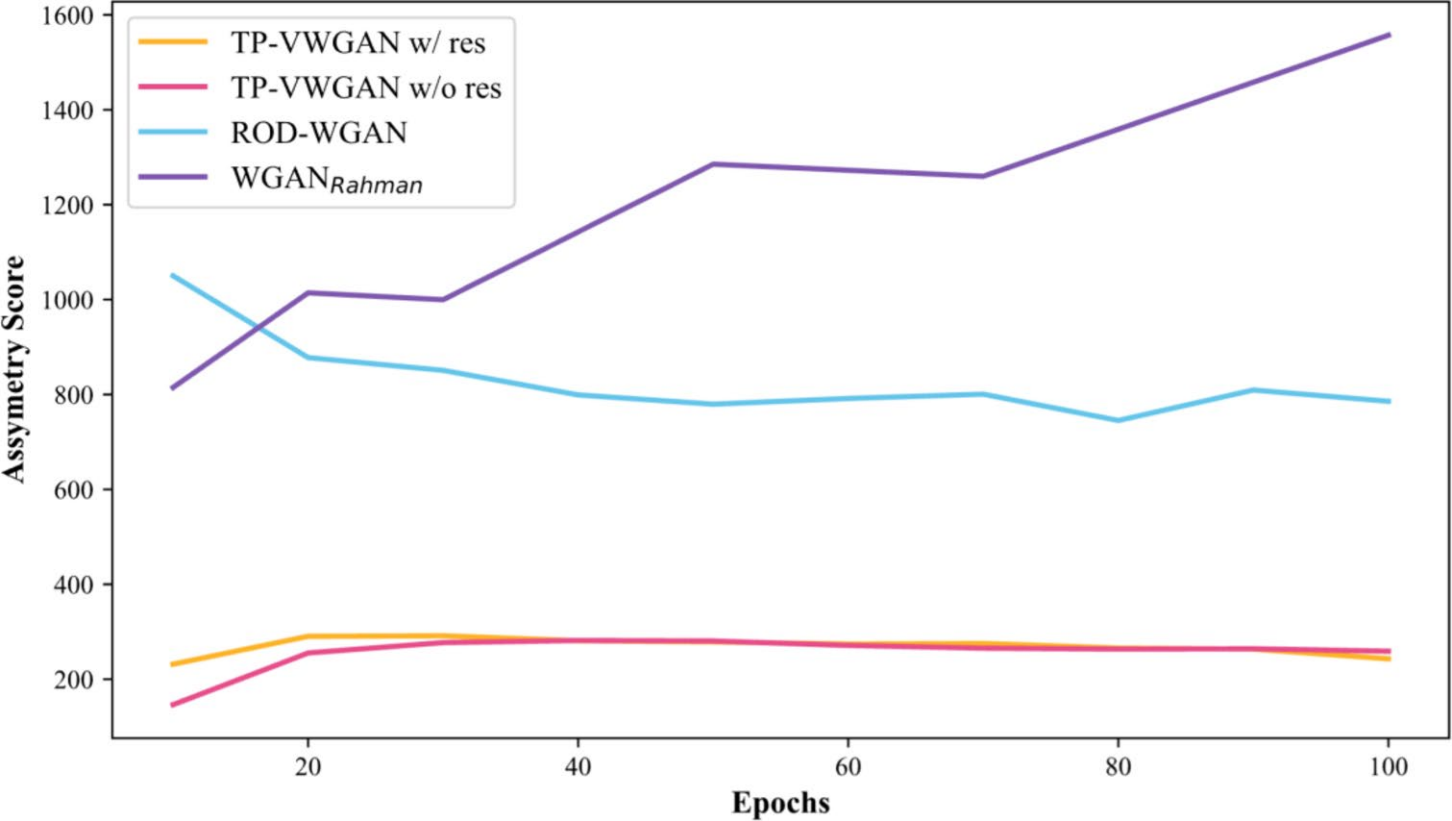
Asymmetry score of the generated distance matrices for different models across training epochs.

Figure 6 tracks the symmetry of the generated distance matrices for different models across epochs. The results show that TP-VWGAN w/ res achieves the lowest asymmetry scores throughout training and is closely followed by TP-VWGAN w/o res. ROD-WGAN performs moderately but has three times the asymmetry of the TP-VWGAN models. WGAN_Rahman_ has the worst symmetry as its asymmetry scores steadily increase. Table 2 summarizes the asymmetry scores at the final epoch and shows this trend: TP-VWGAN w/ res achieves the best symmetry at 243.057, followed by TP-VWGAN w/o res at 258.981, ROD-WGAN at 785.682, and WGAN_Rahman_ at 1557.188. These benchmarks show that models with better symmetry in the generated distance matrices are more effective at capturing key structural features, as confirmed by the evaluations of structural features.

**Table 2:** The final asymmetry scores for different models.

Model	Asymmetry score (final epoch)
TP-VWGAN w/ res	243.057
TP-VWGAN w/o res	258.981
ROD-WGAN	785.682
WGAN_Rahman_	1557.188

### Visualization and analysis of distributions

This subsection visualizes and analyzes the distributions of key structural features in the generated distance matrices by TP-VWGAN at the final epoch. It focuses on comparisons between the generated and real data distributions, evaluates the impact of residual blocks on learning structural features, and assesses the performance of TP-VWGAN relative to ROD-WGAN.

#### Comparison of TP-VWGAN and real distributions

**Fig. 7:**
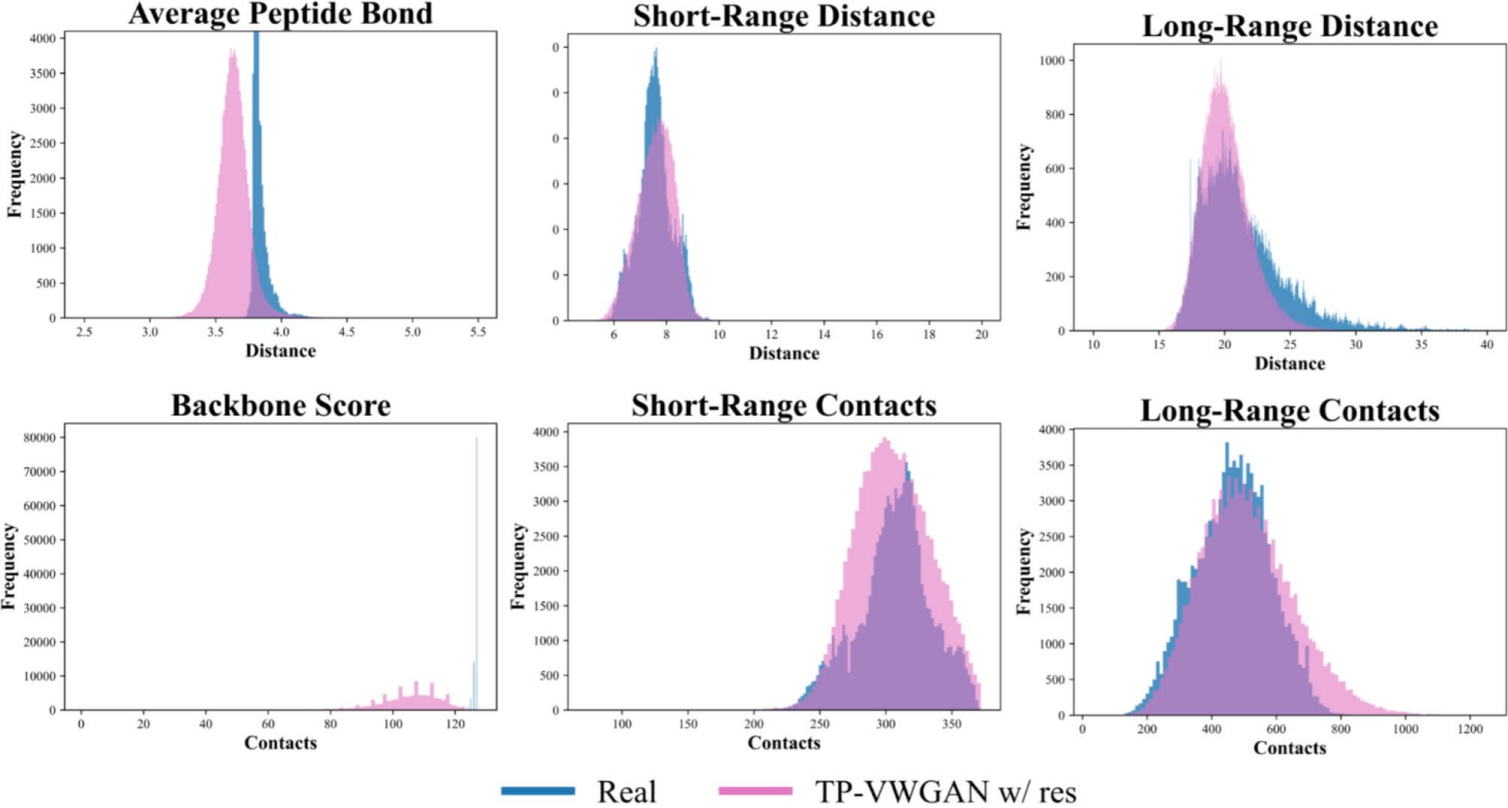
Distributions of key structural features in the distance matrices for TP-VWGAN w/ res and the real dataset at 100 epochs.

Figure 7 shows the backbone, short-range, and long-range structural feature distributions for the real dataset and TP-VWGAN w/ res at the final epoch. The distribution of the generated distance matrices matches the real distributions in both shape and statistical features. For the Average Peptide Bond, TP-VWGAN w/ res shows a mean of 3.640 compared to 3.821 for the real data, with a bit broader spread where the standard deviation is 0.151 compared to 0.050, which points to small differences in variability. The Backbone Score shows a clear shift, with TP-VWGAN w/ res having a lower mean of 105.005 compared to 126.747 for the real data, which indicates some misalignment in the distances between consecutive Cα atoms. The numerical comparisons for these features are summarized in Table 3.

In the Short-Range Distance and Short-Range Contacts distributions, TP-VWGAN w/ res is very similar to real data, with small differences in mean and variance. For Long-Range Distance, TP-VWGAN w/ res shifts to slightly lower values, with a mean of 20.098 compared to 21.364, and has a narrower spread with a standard deviation of 1.863 compared to 3.325. For Long-Range Contacts, it shows higher values, with a mean of 509.377 compared to 464.725 and a wider spread, with a standard deviation of 144.700 compared to 117.042. These results show that TP-VWGAN w/ res generates distance matrices with structural features that mostly match the real distributions but have some differences, especially in the backbone score.

The results also provide evidence of no-mode collapse or overfitting in TP-VWGAN w/ res. The generated distributions are smooth and continuous, without sharp peaks or flat regions, which reflects the model’s ability to capture the full range of structural variations. While the generated distributions approximate the real data, they do not replicate it. For example, in the Average Peptide Bond, the standard deviation for TP-VWGAN w/ res is slightly broader at 0.151 compared to 0.050 for the real data, suggesting that the model generalizes well without simply memorizing the training data. This balance between alignment and variability highlights the model’s ability to generate realistic distance matrices.

The model successfully captures both short-range and long-range structural features, with small differences between the generated and real distributions. The Short-Range Contacts and Short-Range Distance distributions have almost identical means and standard deviations, showing that TP-VWGAN w/ res captures local structural features well. The Long-Range Contacts and Long-Range Distance distributions are also close to the real data but show slight shifts in values. The model slightly overestimates long-range contacts and underestimates long-range distances. These small differences do not affect the model’s ability to generate realistic distance matrices.

**Table 3:** The statistics of key structural features for real data and TP-VWGAN W/ res at 100 epochs.

Structural features	Statistics	Real	TP-VWGAN w/ res
Average peptide bond	Mean	3.821	3.640
	Std	0.050	0.151
Backbone score	Mean	126.747	105.005
	Std	0.632	10.599
Short-range distance	Mean	7.564	7.580
	Std	0.659	0.696
Short-range contacts	Mean	306.352	305.515
	Std	28.313	28.803
Long-range distance	Mean	21.364	20.098
	Std	3.325	1.863
Long-range contacts	Mean	464.725	509.377
	Std	117.042	144.700

#### Effect of residual blocks on TP-VWGAN distributions

**Fig. 8:**
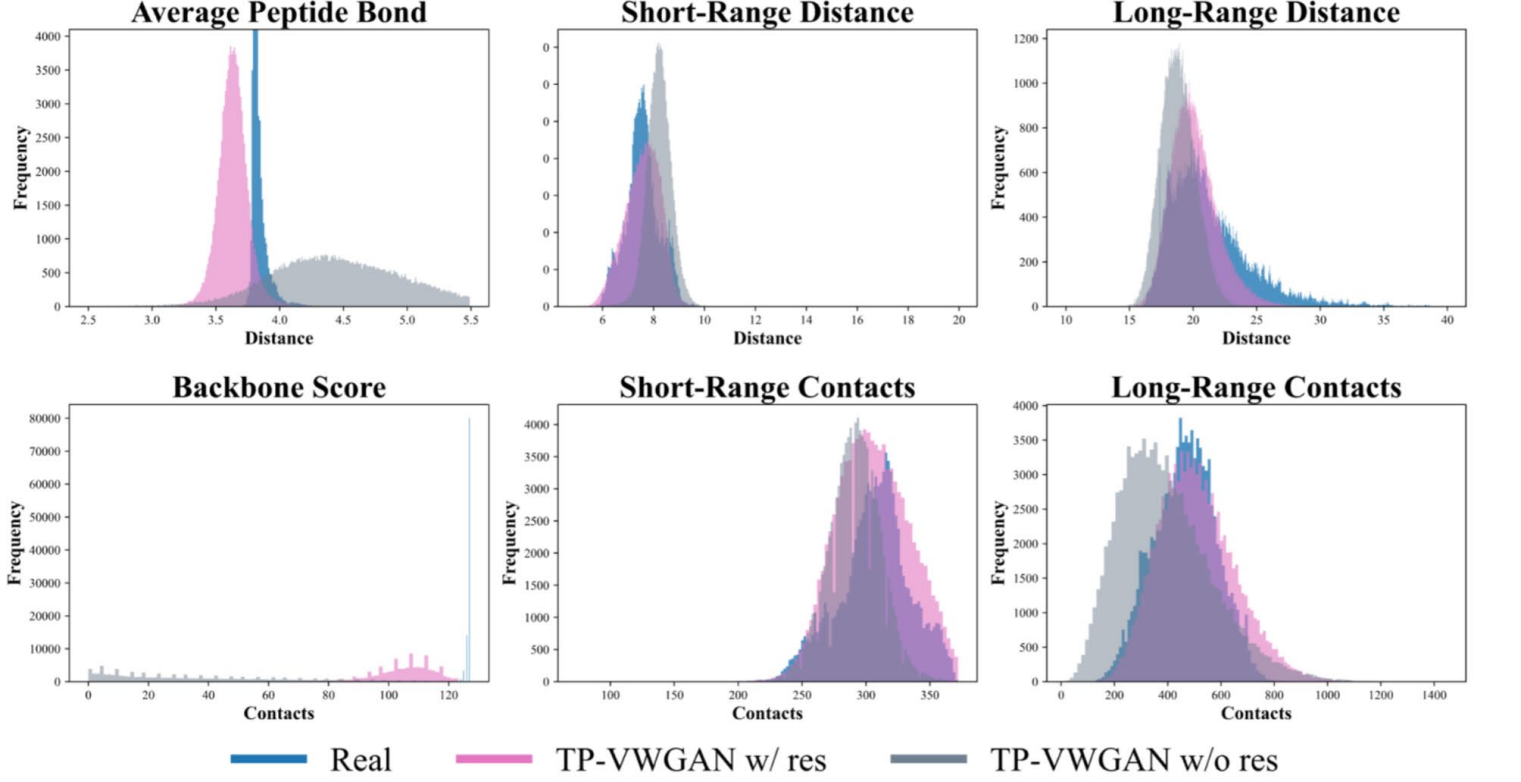
Distributions of key structural features in the distance matrices for TP-VWGAN models with and without residual blocks, and the real dataset at 100 epochs.

Figure 8 presents the structural feature distributions for the real dataset, TP-VWGAN w/ res, and TP-VWGAN w/o res at the final epoch. The generated distance matrices are evaluated for their ability to capture backbone, short-range, and long-range structural features, with numerical comparisons summarized in Table 4.

For the Average Peptide Bond, TP-VWGAN w/ res closely matches the real data, with a mean of 3.640 compared to 3.821 for the real data and a slightly wider spread, with a standard deviation of 0.151 compared to 0.050. TP-VWGAN w/o res shows a much higher mean of 4.489 and a wider spread, with a standard deviation of 0.566, indicating more variability without residual blocks. For the Backbone Score, TP-VWGAN w/ res achieves a mean of 105.005, closer to the real data mean of 126.747. TP-VWGAN w/o res shows a much lower mean of 36.473 and greater variability, with a standard deviation of 29.712 compared to 10.599 for TP-VWGAN w/ res. The results show that adding residual blocks improves consistency and reduces deviations in backbone-related features.

For Short-Range Distance and Short-Range Contacts, TP-VWGAN w/ res performs well, with means and standard deviations nearly identical to the real data. TP-VWGAN w/o res generates higher short-range distances, with a mean of 8.222 compared to 7.580, and fewer short-range contacts, with a mean of 291.228 compared to 305.515 for TP-VWGAN w/ res, with more overall variability. For Long-Range Distance, TP-VWGAN w/ res matches the real data well, with a mean of 20.098 compared to 21.364 and a narrower spread, with a standard deviation of 1.863 compared to 3.325. TP-VWGAN w/o res produces a lower mean of 18.836 and an even narrower spread, with a standard deviation of 1.359, reducing variability in long-range features. For Long-Range Contacts, TP-VWGAN w/ res slightly overestimates the mean, with a value of 509.377 compared to 464.725, and better captures the variability, with a standard deviation of 144.700 compared to 117.042. TP-VWGAN w/o res shows a lower mean of 391.286 and much higher variability, with a standard deviation 175.128.

The comparisons show that TP-VWGAN w/ res generates stable and consistent distance matrices that closely match real data distributions, especially for short- and long-range features. TP-VWGAN w/o res shows larger deviations and less reliable results, particularly for backbone and long-range features. TP-VWGAN w/ res slightly overestimates some features, like long-range contacts, but still generates smoother and more reliable outputs. Residual blocks enhance its ability to create realistic distance matrices with consistent variability.

**Table 4:** Statistical comparison of key structural features in the distance matrices for the real dataset and TP-VWGAN models with and without residual blocks at epoch 100.

Structural features	Statistics	Real	TP-VWGAN w/ res	TP-VWGAN w/o res
Average peptide bond	Mean	3.821	3.640	4.489
	Std	0.050	0.151	0.566
Backbone score	Mean	126.747	105.005	36.473
	Std	0.632	10.599	29.712
Short-range distance	Mean	7.564	7.580	8.222
	Std	0.659	0.696	0.479
Short-range contacts	Mean	306.352	305.515	291.228
	Std	28.313	28.803	20.347
Long-range distance	Mean	21.364	20.098	18.836
	Std	3.325	1.863	1.359
Long-range contacts	Mean	464.725	509.377	391.286
	Std	117.042	144.700	175.128

#### Comparison of TP-VWGAN and ROD-WGAN distributions

**Fig. 9:**
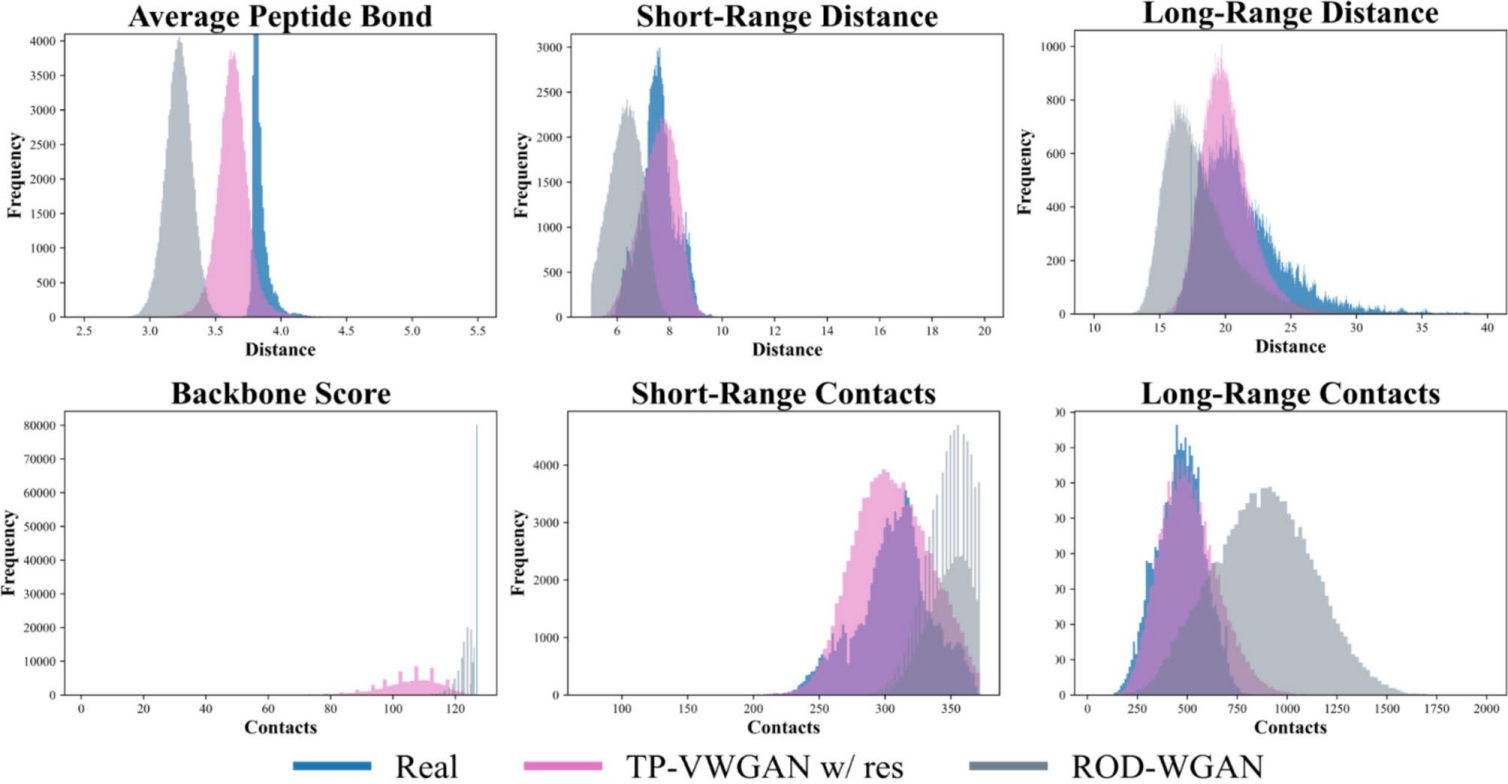
Distributions of key structural features in the distance matrices for TP-VWGAN w/ res, ROD-WGAN, and the real dataset at 100 epochs.

Figure 9 compares the structural feature distributions generated by TP-VWGAN w/ res, ROD-WGAN, and the real dataset at the final epoch. TP-VWGAN w/ res aligns more closely with the real data than ROD-WGAN for most features, as seen in the overlapping distributions. Table 5 provides a summary of the numerical comparisons for the distributions.

For the Average Peptide Bond, TP-VWGAN w/ res has a mean of 3.640, which is closer to the real value of 3.821 than ROD-WGAN’s mean of 3.219. ROD-WGAN also has a narrower spread, with a standard deviation of 0.101 compared to 0.151 for TP-VWGAN w/ res. For the Backbone Score, ROD-WGAN’s mean of 122.819 is closer to the real value of 126.747 than TP-VWGAN w/ res’s mean of 105.005. TP-VWGAN w/ res is closer to the real data in both mean and spread for Short-Range Distance and Short-Range Contacts. ROD-WGAN shows a lower mean for Short-Range Distance, 6.286 compared to 7.564 for TP-VWGAN w/ res, but a higher mean for Short-Range Contacts, 347.447 compared to 306.352 for TP-VWGAN w/ res. For Long-Range Distance and Long-Range Contacts, TP-VWGAN w/ res performs better, with means and variances closer to the real data. ROD-WGAN deviates more, especially in Long-Range Contacts, with a mean of 882.465 compared to 464.725 and a much broader spread, with a standard deviation of 248.969 compared to 144.700.

**Table 5:** Statistical comparison of key structural features in the distance matrices between the real dataset, TP-VWGAN W/ res, and ROD-WGAN at epoch 100.

Structural features	Statistics	Real	TP-VWGAN w/ res	ROD-WGAN
Average peptide bond	Mean	3.821	3.640	3.219
	Std	0.050	0.151	0.101
Backbone score	Mean	126.747	105.005	122.819
	Std	0.632	10.599	2.867
Short-range distance	Mean	7.564	7.580	6.286
	Std	0.659	0.696	0.640
Short-range contacts	Mean	306.352	305.515	347.447
	Std	28.313	28.803	16.178
Long-range distance	Mean	21.364	20.098	18.185
	Std	3.325	1.863	3.024
Long-range contacts	Mean	464.725	509.377	882.465
	Std	117.042	144.700	248.969

### Visualization of generated distance matrices

**Fig. 10:**
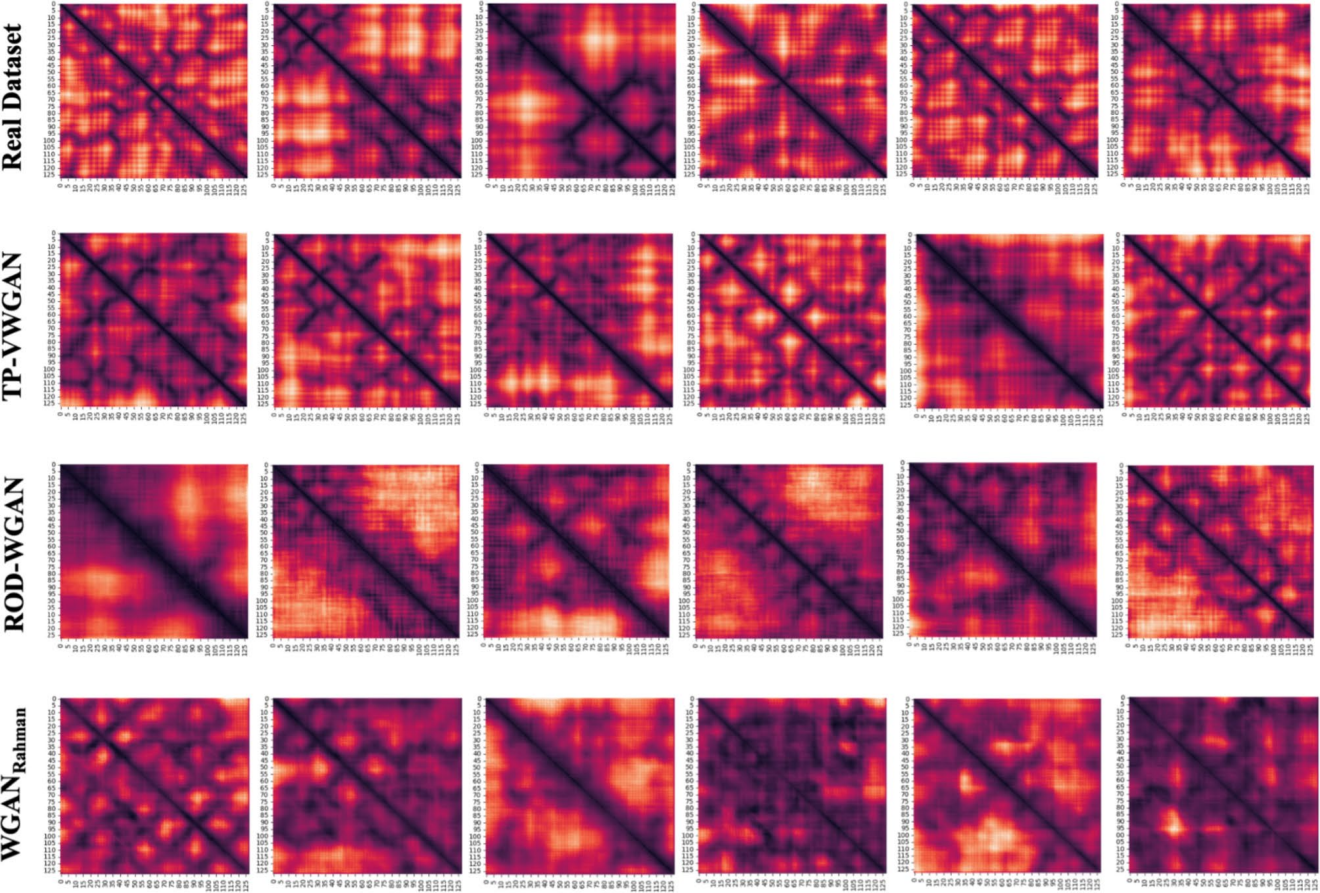
Visualization of randomly selected distance matrices generated by different models (rows), as heatmaps where darker colors represent lower distances.

Figure 10 visualizes random distance matrices generated by different models compared to the real dataset. The first row represents the Real Dataset, serving as the baseline for comparison, while the subsequent rows show matrices generated by TP-VWGAN, ROD-WGAN, and WGAN_Rahman_. Among the models, TP-VWGAN generates the most realistic results, with matrices displaying clear diagonal patterns and smooth structural features that reflect the structural features learned from the real data. TP-VWGAN’s distance matrices demonstrate the ability of the model to generalize key spatial relationships and generate realistic structural distributions. ROD-WGAN generates matrices with blurry, less distinct patterns, which indicates difficulty in learning the spatial patterns in the distance matrices. WGAN_Rahman_ performs the worst, generating highly distorted matrices with missing structural details and uneven diagonal patterns, reflecting a significant deviation from the real data. The visualizations show that TP-VWGAN generates the most realistic and accurate distance matrices, while WGAN_Rahman_ performs the worst.

### Tertiary protein structures generated by TP-VWGAN

**Fig. 11:**
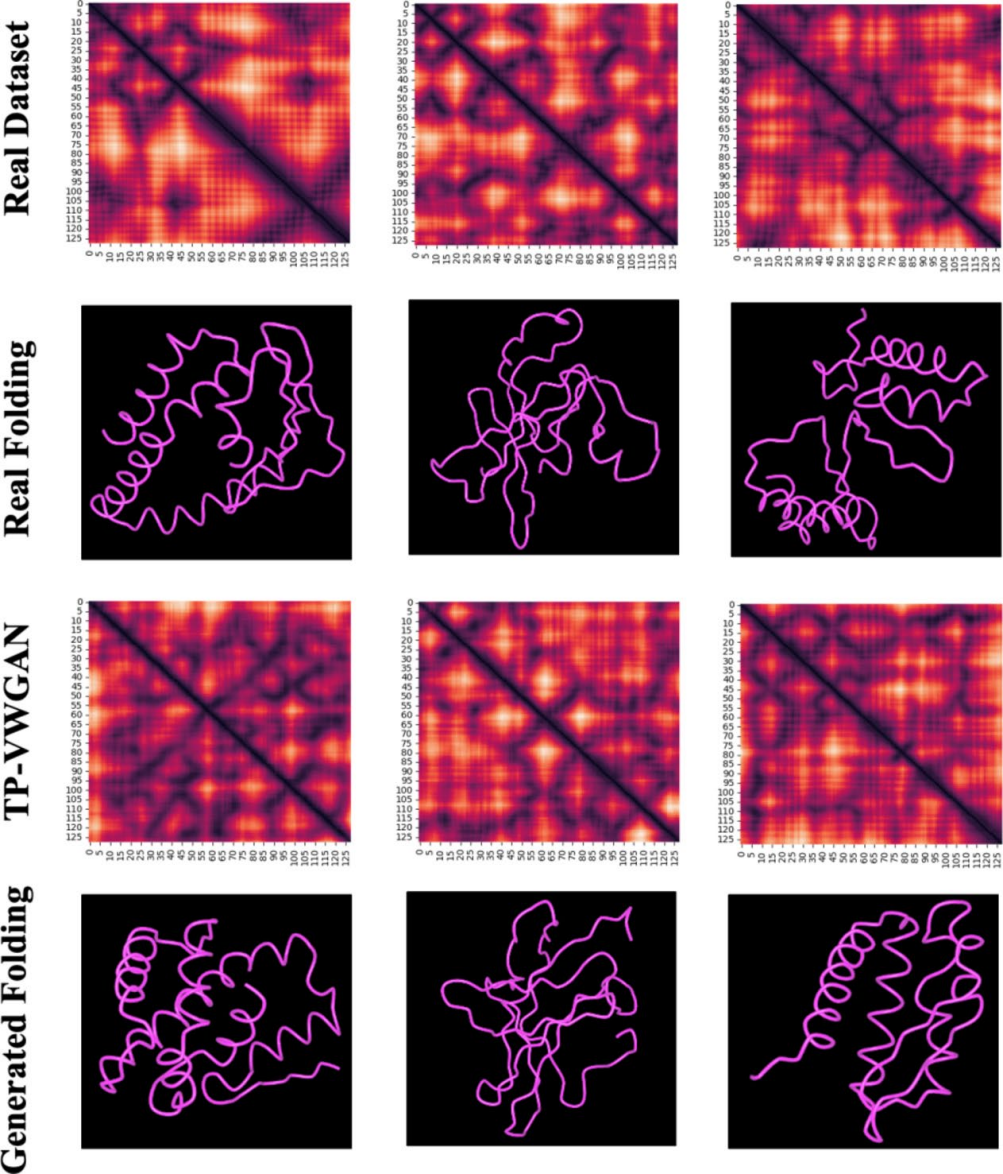
TP-VWGAN generated tertiary protein structures.

Figure 11 shows 3D protein structures based on distance matrices. The visualization includes the distance matrix representation of randomly selected proteins 1a4s, 1acl, and 1aco from PDB, arranged from left to right in the first row, and the second row shows their corresponding 3D folded structures. The third row displays distance matrices generated by TP-VWGAN, while the fourth row illustrates their corresponding generated tertiary structures. These generated structures highlight TP-VWGAN’s ability to capture key patterns in real proteins, such as backbone arrangement and folding, while generating diverse and biologically relevant tertiary conformations. These 3D protein structures were folded using Distance-AF^39^, a modified version of AlphaFold2 that incorporates distance constraints as input information.

## Discussion of results

The proposed TP-VWGAN model, a hybrid of VAE and WGAN-GP, improves the realism of generating protein distance matrices by combining the VAE’s probabilistic learning with the WGAN-GP’s ability to enhance realism. The integration of the residual blocks within the VAE architecture significantly enhanced the model’s performance. The experimental results of the TP-VWGAN, both with and without residual blocks, prove the accuracy of generating realistic protein structures when compared with other existing models; the results also prove the uses of the residual block’s superior performance in capturing key structural features. The main contribution is demonstrating the direct correlation between a model’s ability to learn symmetry features in distance matrices and its ability to capture key structural features accurately.

The evaluation of the TP-VWGAN’s performance was comprehensive, examining both the training dynamics and the model’s generalization capabilities. The convergence analysis, depicted in Fig. 1, demonstrates that the model’s training process was stable and effective. The rapid decline in the reconstruction loss during the initial epochs indicates the decoder’s ability to accurately reconstruct the input distance matrices, suggesting a strong capacity for learning the essential structural features. The stabilization of the Kullback-Leibler divergence (KLD) loss around epoch 20 signifies a well-balanced optimization between the reconstruction accuracy and the latent space regularization. This balance is crucial for ensuring that the model learns a meaningful and well-structured latent representation, which is essential for generating diverse and realistic protein structures.

The structural analysis, which examined the average peptide bond length and backbone score, showed that TP-VWGAN with residual blocks (TP-VWGAN w/ res) consistently outperformed the other models, achieving the lowest MMD, EMD, and BD values and aligning most closely with real data. TP-VWGAN without residual blocks (TP-VWGAN w/o res) also performed well but had slightly higher deviations. Focusing on short-range distances and contacts analysis also highlighted the strength of TP-VWGAN w/ res, which again achieved the lowest MMD, EMD, and BD values. TP-VWGAN w/o res followed closely but showed slightly higher deviations, especially in MMD, while ROD-WGAN showed larger variations and instability, indicating weaker performance. WGAN_Rahman_ consistently performed the worst, with the highest and most unstable values across all metrics. The long-range structure analysis reinforced these findings, as TP-VWGAN w/ res continued to deliver the best results with the lowest and most stable values. TP-VWGAN w/o res remained competitive but showed slightly higher deviations in MMD and EMD, while ROD-WGAN demonstrated moderate alignment with stable yet higher values. WGAN_Rahman_ remained the weakest performer, showing the highest and least stable values. These results confirm that TP-VWGAN w/ res effectively captures structural features, which are crucial for accurately modeling protein tertiary structures.

The experimental results show a significant correlation between symmetry scores and the evaluation of structural features, highlighting the importance of symmetry in capturing meaningful structural information. Models that demonstrate better symmetry in their generated distance matrices are more effective at accurately representing the key structural features. This correlation validates symmetry analysis as a useful metric for assessing the quality of learned representations.

A comparative analysis between TP-VWGAN with and without residual blocks reveals the crucial role of these blocks in enhancing model performance. The version with residual blocks (TP-VWGAN w/ res) aligns more closely with real data distributions, but the version without residual blocks shows larger variances, particularly in backbone and long-range features.

The visualization of generated distance matrices of TP-VWGAN, as shown in Fig. 10, demonstrates its ability to generate highly realistic results with clear diagonal patterns and smooth structural features that align with real data. These matrices reflect the model’s capacity to generalize key spatial relationships and generate accurate structural distributions. ROD-WGAN generates blurry matrices with less distinct patterns, suggesting difficulties in capturing spatial structures. WGAN_Rahman_ performs the worst, generating highly distorted matrices with missing structural details and uneven diagonal patterns, indicating a significant deviation from real data. These visual differences reinforce that TP-VWGAN achieves the highest realism while WGAN_Rahman_ struggles the most.

## Conclusion and future work

This paper presented TP-VWGAN, a hybrid model that combines the probabilistic representation learning of a Variational Autoencoder (VAE) with the adversarial training of a Wasserstein Generative Adversarial Network with Gradient Penalty (WGAN-GP) to improve the realism of generating distance matrix representations of tertiary protein structures. The model incorporated residual blocks into its VAE architecture to enhance performance, and these enhancements were validated by comparing the model with and without the residual blocks. Thorough evaluation using several metrics that assess the presence of backbone, short-range, and long-range structures demonstrated that TP-VWGAN generates tertiary protein structures with the same physical realism as the training dataset, showing effective learning, generalization, and better performance compared to existing methods while demonstrating that the more accurately a model learns symmetry features in the generated distance matrices, the better it captures key structural features in tertiary protein structures.

The results show that TP-VWGAN represents a step forward in generative models for protein structure modeling. However, using only Protein Data Bank (PDB) data may limit its ability to handle complex protein structures. Future work will focus on expanding the training dataset with protein structures from additional repositories to improve generalization. Enhancements will also include generating tertiary protein structures using both distance and dihedral angle representations to increase structural realism. Additional efforts will aim to adapt the model for proteins of varying lengths and explore broader applications of generated data.

## Methodology

This section describes the dataset used for training the model, followed by a detailed explanation of the methodology and architecture. It also presents the evaluation metrics used to assess the generated data, along with the hardware and software specifications and model hyperparameters used in the experiments.

### Dataset

**Fig. 12:**
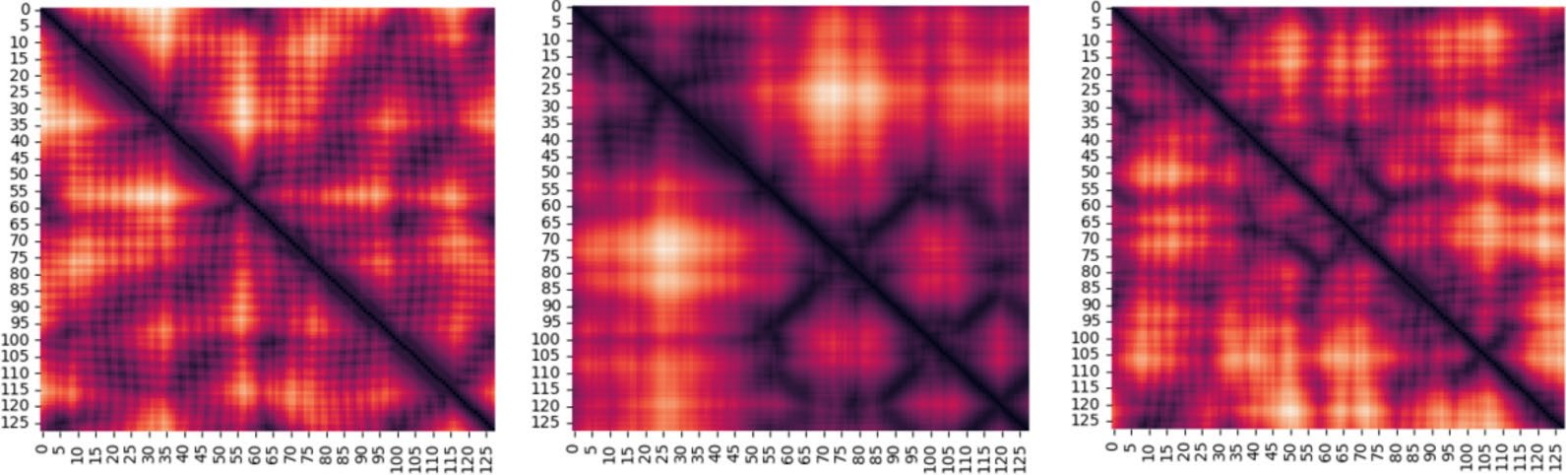
Samples of distance matrices from the training data.

This paper uses a dataset from the PDB^14^ of 122,082 full-atom protein structures, each with varying amino acid lengths. The dataset is prepared by extracting non-overlapping fragments of 128 aa from each protein structure. Each fragment is converted into a distance matrix of size 128 × 128, where each entry records the Euclidean distance between the Cα atoms of two residues. The resulting matrices are symmetric because the distance between two residues is always the same, no matter which way it is measured. This transformation encodes the 3D full-atom structure of a protein molecule into a 2D distance matrix and offers a simplified representation for computational analysis. The training dataset consists of 98,768 distance matrices sampled from the full dataset, while the testing dataset includes 7,069 distance matrices derived from 6,247 protein structures excluded from training. Figure 12 shows samples of the distance matrices from the training data.

Using 128 aa fragments ensures a balanced representation of protein structures, as the length matches the average amino acid sequence in the dataset. A fragment of size 128 is sufficient to capture key structural features without being too short, which could fail to capture interactions between residues that are moderately spaced apart. Smaller fragments can also overuse parts of the same protein, which lowers diversity in the dataset. Larger fragments result in fewer fragments being extracted from each protein, which means some structures may be excluded, and the dataset becomes smaller.

### The proposed methodology

The TP-VWGAN integrates the encoder and decoder of a VAE with the critic of a WGAN-GP to generate distance matrices of protein tertiary structures, each having a distinct role during training. Residual blocks are incorporated into the VAE architecture to improve the model’s ability to capture key structural features. These blocks use skip connections to pass information directly across layers, which makes it easier for the network to learn refinements or residual mappings instead of starting from scratch at each layer. This enhancement avoids vanishing gradients, speeds up training, and improves the model’s capacity to represent complex patterns in distance matrices. Pseudocode 1 outlines the main steps of the training methodology, showing how the encoder, decoder, and critic work together.

**Fig. 13:**
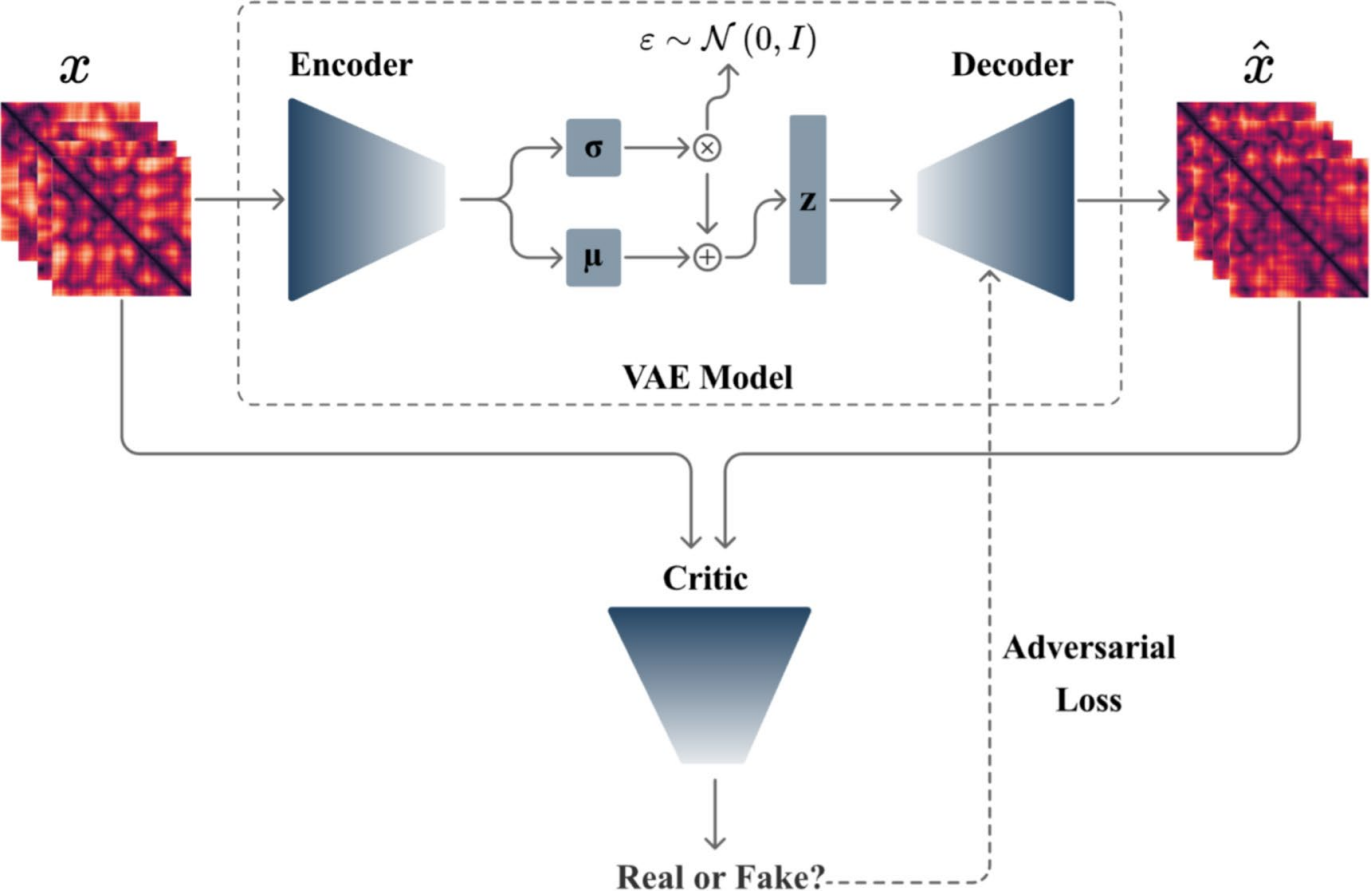
Main components of the methodology.


Figure 13 shows the main components of the methodology used to generate distance matrices. The training process begins by feeding input data $\:x$ into the encoder, which processes it into a latent vector $\:z$ characterized by a mean $\:\mu\:$ and standard deviation $\:\sigma\:$. The latent vector $\:z$ is then passed to the decoder, which reconstructs the input data $\:x$ as $\:\hat x$. During training, the encoder and decoder update their parameters to minimize the VAE loss, which includes both a reconstruction term and a regularization term. The regularization term used in the methodology is the Kullback-Leibler Divergence (KLD), which helps ensure that the latent space follows a standard normal distribution.

After minimizing the VAE loss, the training process shifts to refining the critic and decoder to improve the realism of the generated data. The decoder is treated as the generator in WGAN-GP, which generates data to challenge the critic. The critic is updated iteratively by calculating a loss that measures the difference between its evaluations of real and generated (fake) data, with an added gradient penalty term to ensure stability. Minimizing this loss makes the critic more effective at distinguishing real data from fake data. Once the critic is refined, fake data is generated again by passing noise through the decoder. The adversarial loss is then computed based on the critic’s evaluation of this fake data. The decoder is updated to minimize this adversarial loss, enhancing its ability to produce realistic data that aligns with the critic’s expectations.

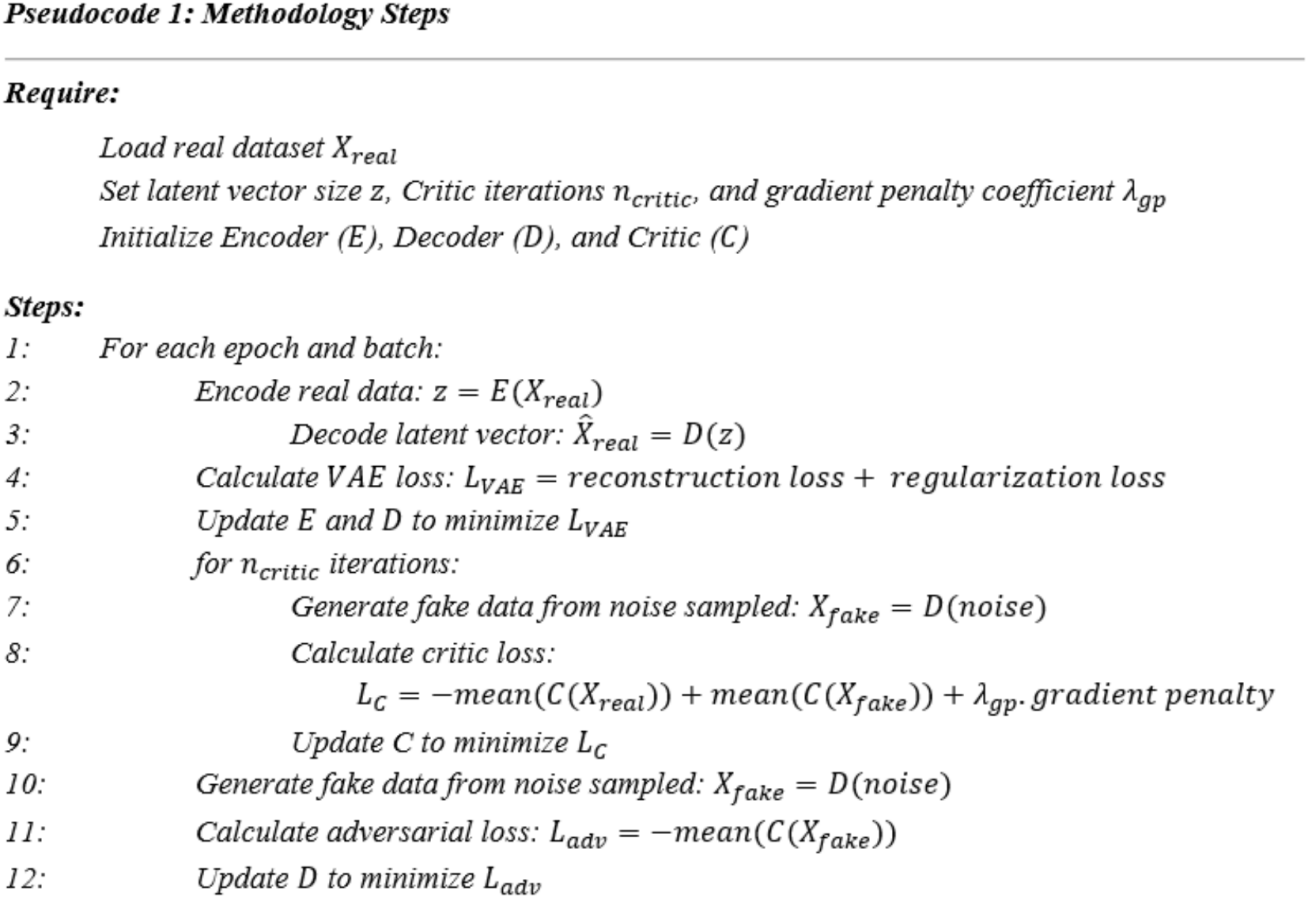


#### VAE architecture

**Fig. 14:**
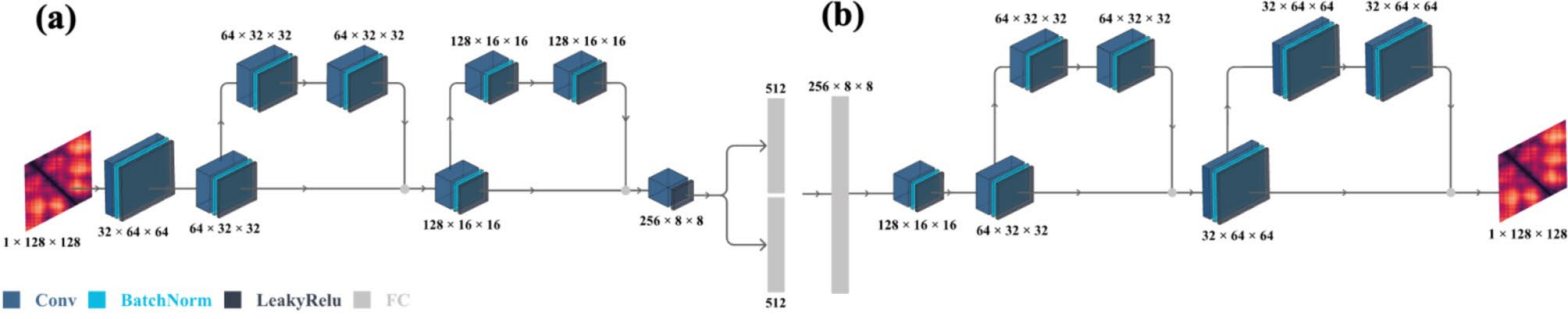
Architecture of the convolutional VAE for processing 128 × 128 distance matrices: (**a**) the encoder network, (**b**) the decoder network.

The VAE used in the methodology comprises an encoder and a decoder. The encoder utilizes a series of convolutional layers and residual blocks to progressively reduce spatial dimensions and extract critical features, compressing the input into a latent representation. The decoder reconstructs the data using transposed convolutional layers and residual blocks, restoring the spatial dimensions to their original size. All layers in the encoder and decoder, except the final transposed convolutional layer in the decoder, incorporate Batch Normalization and are activated by the Leaky ReLU function, which stabilizes training and enhances feature extraction. The detailed architecture of the VAE is provided in Tables 6 and 7 and illustrated in Fig. 14 for input dimensions of 128 × 128 distance matrices.

The encoder begins with an input layer for images of dimensions 1 × 128 × 128. The first convolutional layer with 32 filters of size 4 × 4, stride 2, and padding 1 reduces the spatial dimensions to 64 × 64. Following the first convolutional layer, a 64-filter convolutional layer further downscales the dimensions to 32 × 32. A residual block, consisting of two 64-filter convolutional layers and a skip connection, is then applied to refine features. Additional convolutional layers reduce the dimensions to 16 × 16 through a 128-filter convolutional layer and another residual block with 128 filters. The encoder concludes with a 256-filter convolutional layer that outputs a feature map 256 × 8 × 8, which is flattened and passed to two fully connected layers to generate the mean vector µ and log variance log σ^2^ in a 512-dimensional latent space.

Following the encoder’s compression for the input data, the decoder begins the reconstruction process by upsampling the latent vector from 512-dimensional space. A fully connected layer projects the latent vector into a feature map of dimensions 256 × 8 × 8. Transposed convolutional layers with 256, 128, 64, and 32 filters then progressively restore the spatial resolution. Residual blocks with 64 and 32 filters are included after the second and third transposed convolutional layers to preserve feature continuity. The final transposed convolutional layer, with 32 filters of size 4 × 4, reconstructs the image to its original dimensions of 1 × 128 × 128.

**Table 6:** Layers of the encoder network architecture.

Layer		Dimensions	Filter	Stride	Padding
Input		1 × 128 × 128	–	–	–
Convolutional 1		32 × 64 × 64	32 × 4 × 4	2	1
Convolutional 2		64 × 32 × 32	64 × 4 × 4	2	1
Residual block 1	Convolutional 1	64 × 32 × 32	64 × 3 × 3	1	1
	Convolutional 2	64 × 32 × 32	64 × 3 × 3	1	1
Convolutional 3		128 × 16 × 16	128 × 4 × 4	2	1
Residual block 2	Convolutional 1	128 × 16 × 16	128 × 3 × 3	1	1
	Convolutional 2	128 × 16 × 16	128 × 3 × 3	1	1
Convolutional 4		256 × 8 × 8	256 × 4 × 4	2	1
Fully connected		512	–	–	–
Fully connected		512	–	–	–

**Table 7:** Layers of the decoder network architecture.

Layer		Dimensions	Filter	Stride	Padding
Input		512	-	-	-
Fully connected		256 × 8 × 8	-	-	-
Deconvolutional 1		128 × 16 × 16	256 × 4 × 4	2	1
Deconvolutional 2		64 × 32 × 32	128 × 4 × 4	2	1
Residual block 1	Convolutional 1	64 × 32 × 32	64 × 3 × 3	1	1
	Convolutional 2	64 × 32 × 32	64 × 3 × 3	1	1
Deconvolutional 3		32 × 64 × 64	64 × 4 × 4	2	1
Residual block 2	Convolutional 1	32 × 64 × 64	32 × 3 × 3	1	1
	Convolutional 2	32 × 64 × 64	32 × 3 × 3	1	1
Deconvolutional 4		1 × 128 × 128	32 × 4 × 4	2	1

#### Critic architecture

**Fig. 15:**
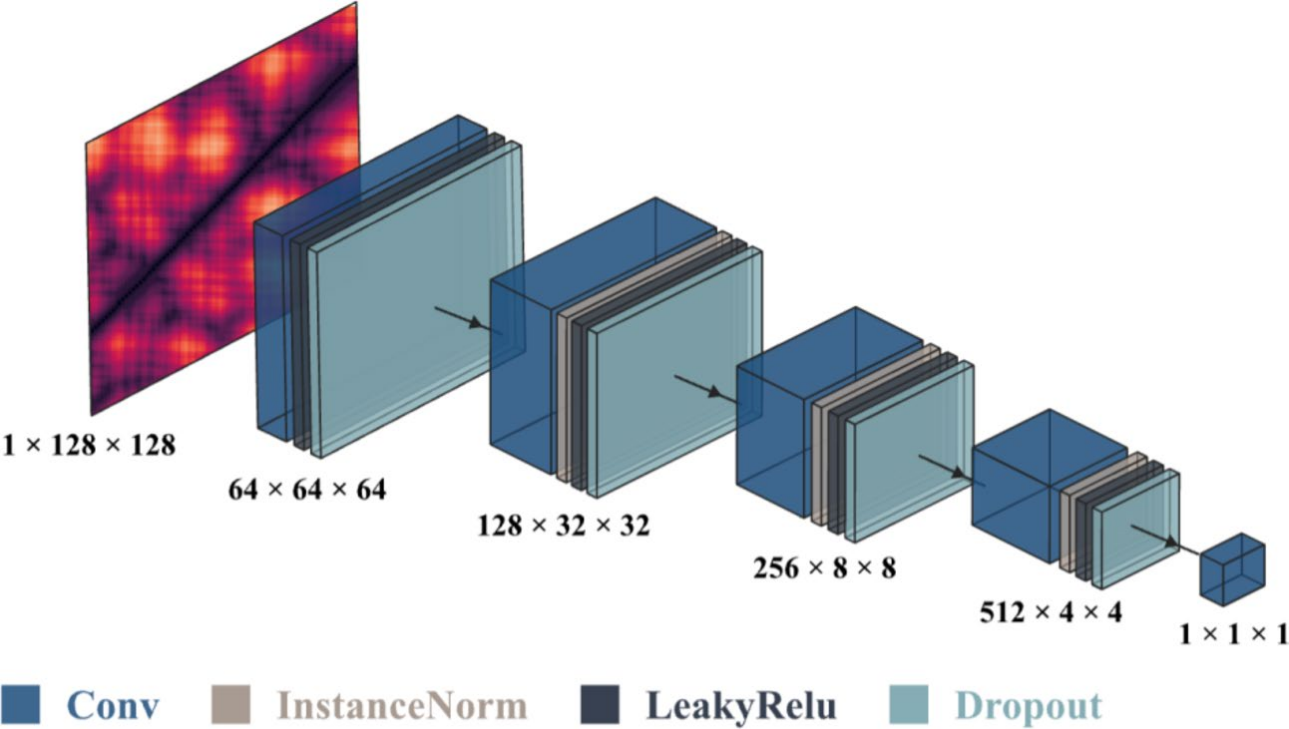
Critic architecture for 128 × 128 distance matrices.

The critic has five convolutional layers that gradually reduce the spatial dimensions of the input, outputting a single scalar value, which evaluates the likelihood of the matrix being real and guides the refinements of the decoder during adversarial training. The detailed architecture of the critic is provided in Table 8 and illustrated in Fig. 15. Figure 15 shows the Critic architecture for 128 × 128 distance matrices.

The first convolutional layer in the critic uses 64 filters with a kernel size of 4 × 4, a stride of 2, and padding of 1, coupled with Leaky ReLU activation and Dropout for regularization but no normalization. The number of filters increases to 128, 256, and 512 in subsequent layers, which apply Instance Normalization, Leaky ReLU, and Dropout to stabilize training and prevent overfitting. The final convolutional layer, with one filter of size 4 × 4, no Instance Normalization, and no Dropout, outputs a single scalar value to evaluate the input matrix.

**Table 8:** Layers of the critic network architecture.

Layer	Dimensions	Filter	Stride	Padding	Dropout
Input	1 × 128 × 128	–	–	–	–
Convolutional 1	64 × 64 × 64	64 × 4 × 4	2	1	0.1
Convolutional 2	128 × 32 × 32	128 × 4 × 4	2	1	0.1
Convolutional 3	256 × 8 × 8	256 × 4 × 4	4	1	0.1
Convolutional 4	512 × 4 × 4	512 × 4 × 4	2	1	0.1
Convolutional 5	1 × 1 × 1	1 × 4 × 4	1	0	–

### Evaluation of the generated distance matrices

This subsection describes the metrics used to evaluate the realism of the generated distance matrices: backbone, short-range, and long-range structure. Each feature provides insights from two perspectives: contact counts and average distances. This paper uses all these perspectives, based on approaches found in related work, for a thorough evaluation. The evaluation also includes different metrics to compare the distributions of these features between real and generated distance matrices and an assessment of matrix asymmetry.

#### Evaluation of structural features

The generated distance matrices are assessed for the presence of the backbone, short-range, and long-range structural features using specific metrics^25,26^. As summarized below, each metric reduces the distance matrix to a single number.

##### Backbone structure

The backbone structure is evaluated by analyzing consecutive Cα atom pairs $\:(i,\:i+t)$. The Average Peptide Bond Length calculates the mean distance between these pairs, while the Backbone Length Score counts how many of these pairs are within a distance of 4 Å.

##### Short-range structure

The short-range structure involves Cα pairs $\:(i,\:i+t)$ where $\:1<t\le\:4$ (the backbone pairs are not included here). The Short-Range Contacts metric counts the number of such pairs within a distance of 10 Å, and the Short-Range Distance computes their average distance.

##### Long-range structure

Cα pairs $\:(i,\:i+t)$, where $\:t>4$, represent the long-range structure. Similar to the short-range, the Long-Range Contacts metric counts how many of these pairs are within 10 Å, and their average distance is referred to as the Long-Range Distance.

#### Comparison of distributions

After evaluating the structural features, the next step is to compare the distributions of these features between the generated and real distance matrices. Three metrics are used for this comparison: Maximum Mean Discrepancy (MMD)^40^, Earth Mover’s Distance (EMD)^41^, and Bhattacharyya Distance (BD)^42^. The rationale for using multiple metrics is that each evaluates the distributions from a different perspective. MMD measures the largest difference between the expected values of the distributions, focusing on overall dissimilarity. EMD, or Wasserstein distance, calculates the minimum cost of transforming one distribution into another, considering the amount moved and the distance traveled. BD measures the similarity between two distributions by quantifying how much they overlap.

#### Matrix asymmetry evaluation

The symmetry in the generated distance matrices was found to correlate with the model’s ability to capture key structural features. The Frobenius Norm^43^ was used to measure the asymmetry of the distance matrix, as shown in Eq. (1):

1 $$\:Asymmetry\:Score\:={\left|D-{D}^{T}\right|}_{F}=\sqrt{{\varSigma\:}_{i=1}^{m}{\varSigma\:}_{j=1}^{n}{\left|{d}_{ij}-{d}_{ji}\right|}^{2}}\:$$

where $\:D$ is a distance matrix, and $\:{D}^{T}$ is its transpose.

### Hardware and software specifications

The implementation was carried out using Python 3.12 and PyTorch, running on an NVIDIA RTX 4060 GPU with CUDA 11.8 and an AMD Ryzen 5 7600 processor running at 3.80 GHz. The setup was used to train and evaluate the model.

### Methodology hyperparameters

The methodology was trained using a learning rate of $\:{10}^{-4}$ for the encoder, decoder, and critic, using the Adam optimizer ($\:{\beta\:}_{1}=0.5$ and $\:{\beta\:}_{2}=0.999$). A gradient penalty coefficient $\:{\lambda\:}_{gp}$ of 10 ensured the Lipschitz constraint, with the critic updated 5 times per generator update $\:{n}_{critic}=5$. The training was conducted over 100 epochs with a batch size of 64, and the latent space dimensionality was set to 512.

## Data Availability

All data used during this study are cited in this published article.
